# Mn-modified phosphomolybdates by hydrothermal route for pseudocapacitor application

**DOI:** 10.1371/journal.pone.0346559

**Published:** 2026-04-08

**Authors:** Nusrat Tazeen Tonu, Mohammad Abu Yousuf

**Affiliations:** 1 Department of Chemistry, Khulna University of Engineering and Technology, Khulna, Bangladesh; 2 Chemistry Discipline, Khulna University, Khulna, Bangladesh; Tohoku University, JAPAN

## Abstract

In this study, hydrothermal method was used to synthesize Mn modified organic-inorganic phosphomolybdates, Product-1 and 2, as considered to be, [Mn_x_(Imi)_y_{PMo_12_O_40_}], [Mn_x_(Imi)_y_{P_2_Mn_2_Mo_12_O_50_}] with Product-3, which was considered to be mixed by-products, might have [Mn_x_(Imi)_y_{P_2_Mn_2_Mo_12_O_50_(Imi)_4_Na_4_(OH)_8_}(OH)_z_] (majority) in its structure, (imi = imidazole). Product-1 was the main product, Product-2 and 3 were prepared from the by-product using only heating followed by concentrating technique. All products had high temperature resistance. According to XPS data, the percentages of Mo, Mn, C, O, P, N and Na were 3.91, 23.30, 13.54, 47.83, 7.9, 2.30, and 1.15%, 20.10, 2.39, 22.98, 41.53, 3.53, 6.83, and 2.6%, and 15.51, 1.06, 31.29, 43.56, 1.84, 5.58, and 0.58%, in Product-1, 2 and 3, respectively (by the no. of atoms), along with the average valency states of Mn were 2.26, 2.39, and 2.14, respectively. Product-1 was considered having polyoxo kegging clusters, Product-2 and 3 were considered having polyoxohexamolybdate units in their structures, as well as Product-2 was deliberately assumed to be the polymer or a derivative of Product-3. Cyclic voltammetry (CV) showed all three compounds having different reversible pseudo-capacitor character. At 2.0 Ag^-1^ current density, Product-1, 2 and 3@GCE showed specific discharge capacitance of 37.01, 380.45, and 635.37 Fg^-1^, respectively. In galvanostatic charging-discharging (GCD), Product-1@GCE showed comparatively higher retention as compared to other electrodes.

## Introduction

Energy is an essential global commodity and has become a key element for worldwide progress. Due to the consumption of traditional energy resources by population growth, the need of sustainable energy storage is rising day by day. Thus the development of new energy storage device has been indicated as one of the top goals for researchers [1–2]. Supercapacitors, a kind of energy storage devices, are intermediate between conventional capacitors and batteries, and are gaining popularity because of their rapid charging-discharging, high power density, long cycle retention, high charge-discharge efficiency, and long operating lifetime [3–4]. There are two main energy storage mechanisms of supercapacitors, electric double layer capacitor (EDLC) and pseudocapacitance (PC). EDLC stores electric charge through electric double layers (EDL) at electrode/electrolyte interface. Carbon-based materials, such as carbon nanotube, activated carbon, graphene, carbon aerogel, etc. exhibit EDLC mechanism. In this case, a voltage is applied to the electrodes, which migrate ions from the electrolyte to the surface of electrodes, creating EDL, and energy is stored, which is a completely physical adsorption-desorption process and no electrochemical reaction happened at the electrode surface [5–6]. On the contrary, pseudocapacitors store energy through Faradic or electrochemical redox reactions on the surface of the electrode [7].

Among a variety of pseudocapacitors, polyoxometalate (POM) – based complex compounds are gaining their popularities due to their ironic structure, thermal strength, and redox activity. Besides, they have the ability to change their structure by coordination with different ligands and foreign metals, in so doing changing themselves from low to high conductive water (in)soluble polymers [8–9]. POMs are self-assembled multinuclear nanoclusters which consists high valent transition metals from group VB (Vanadium, V) or group VIB (Molybdenum, Mo or tungsten, W) and they are negatively charged, consist three or more transition metal oxyanions with shared oxygen atoms, forming a large closed 3D-framework [10–11]. Generally, POMs are group of anionic inorganic clusters having general formula [X_x_M_m_O_y_]^n-^, where, M is addenda atom which is used to make the 3D-framework or sole structure (usually d-block element, V^V^, Mo^VI^ or W^VI^) and can be substituted by metal ions from almost all regions of the periodic table (s-, p-, d-, and f-block), X is a heteroatom (generally main group element, P, Si, Ge, As) are enclosed in the center of the framework, which also share adjacent oxygen atoms with transition metal addenda ions [12–13].

The open framework of POM consists independent structural units having limited or single electron transfer properties, low electronic conductivity, and susceptible to to dissolve in many electrolytes, which limits its use in aqueous energy storage system [14–15]. To overcome this problem, POMs are usually modified by coordination to other transition metals and ligands, which alters its electronic structure of main cluster, thereby changes its solubility, increases its surface area, thermal rigidity and conductivity as well [16–17]. POMs modification follows six types of fundamental structure of POMs, e.g., Kegging, Anderson, Waugh, Lindqvist, Silverton, and Dowson [18–19]. The most common techniques of modification involved hydrothermally removal of one or more addenda atom (Mo) sites from kegging anions, resulting [PMo_12_O_40_]^4-^ to [PMo_11_O_39_]^8-^ + MoO^4+^, in which empty binding sites are available for the addition of new metal ions (Fe, Cu, Mn, etc.) and ligands for further functionalization [20–21]. This modification of POM cluster to kegging cluster directs POMs to thermal stability, larger molecular sizes and weights, non-toxicity, non-volatility, non-solubility in oxygenated organic solvents (water, acetone, ethanol, ether, etc.), robustness in redox properties, change of color, and effective polymerization to metal-organic-inorganic skeleton [22–23]. Through this action, the selection of ligand is also very important for having electrochemically flexible host type structure for capacitive application. Flexible organic amines are very popular for their flexibility in coordination bonding, such as bis(triazole), bis(pyridine), bis(Pyrazole), etc. [24–25].

Xingzhi, *et al.*, introduced different metal-organic units into [PMo_12_]-POM system and synthesized three porous coordination keggin polymers, $\left\{\text{Cu}(\text{pra}{)}_{\text{2}}\}[{\left\{\text{Cu}(\text{pra}{)}_{\text{2}}\right\}}_{\text{3}}\left\{\text{P}\overset{VI}{\underset{\text{11}}{\text{Mo}}}{\text{Mo}}^{VI}O_{\text{4}\text{0}}\right\}\right]$, $\left[\left\{{Ag}_{\text{5}}(\text{pz}{)}_{\text{6}}{H_{\text{2}}O}_{\text{0}.\text{5}}\text{Cl}\right\}\left\{\text{P}\overset{VI}{\underset{\text{11}}{\text{Mo}}}{\text{Mo}}^{VI}O_{\text{4}\text{0}}\right\}\right]$, and $\left[\left\{{Cu}_{\text{3}}(\text{bpz}{)}_{\text{5}}H_{\text{2}}O\right\}\left\{\text{P }{Mo}_{\text{1}\text{2}}O_{\text{4}\text{0}}\right\}\right]$; (where, pre = Pyrazole, pz = pyrazine, and bpz = benzopyrazine, through hydrothermal route), which showed large capacitances 672.2, 782.1, and 765.2 Fg^-1^, respectively, at current density 2.4 Ag^-1^, with superior cycling retention of 91.5, 89.3, and 87.8%, respectively, over 5000 cycles [10]. Hongtao, et al., also obeyed hydrothermal method and prepared four kegging-based complexes, ${[Zn}_{\text{4}}\left({Ccbypy)}_{\text{1}\text{0}}{\left(H_{\text{2}}O\right)}_{\text{4}}{\left(Si{Mo}_{\text{1}\text{2}}O_{\text{4}\text{0}}\right)}_{\text{2}}\right].\text{8}H_{\text{2}}O$, ${[Cu}_{\text{2}}\left({Ccbypy)}_{\text{4}}{\left(H_{\text{2}}O\right)}_{\text{2}}Si{Mo}_{\text{1}\text{2}}O_{\text{4}\text{0}\;}\right].\text{3}H_{\text{2}}O$, ${[Zn}_{\text{4}}\left({Ccbypy)}_{\text{1}\text{0}}{\left(H_{\text{2}}O\right)}_{\text{4}}{\left(SiW_{\text{1}\text{2}}O_{\text{4}\text{0}}\right)}_{\text{2}}\right].\text{6}H_{\text{2}}O$, and ${[Cu}_{\text{2}}\left({Ccbypy)}_{\text{4}}{\left(H_{\text{2}}O\right)}_{\text{2}}SiW_{\text{1}\text{2}}O_{\text{4}\text{0}\;}\right].\text{4}H_{\text{2}}O$, where, Ccbupy = 4-Carboxy-1-(2´-cyano-biphenyl-4-ylmethyl)-pyridinium, and found extreme capacitance (1520.8 Fg^-1^ at 1.0 Ag^-1^ current density) of the 2^nd^ one compound with cycle stability of 96.61% after 1000 cycles of charging-discharging compared to other three compounds [26]. Dongfeng, *et al.*, developed two Mo-based POM by Cu and asymmetric N-containing ligands and observed high specific capacitances (249.0 and 154.5 Fg^-1^, at 3.0 Ag^-1^ current density) [27]. Dongfeng, *et al.*, further employed hydrothermal route and synthesized five Cu-containing Mo-based POM Keggin complexes, among them $\left[\overset{I}{\underset{\text{4}}{Cu}}H_{\text{2}}{\left(btx\right)}_{\text{5}}{\left(P{Mo}_{\text{1}\text{2}}O_{\text{4}\text{0}}\right)}_{\text{2}}\right].{\text{2}H}_{\text{2}}O$, where, btx = 1,4-bis(triazole-1-ylmethyl)benzene) exhibited the highest specific capacitance 237.0 Fg^-1^ at 2.0 Ag^-1^ current density, and 92.5% retention after 1000 cycles of GCD [28]. In this work, a simple hydrothermal route was adopted to synthesize manganese acetate and imidazole modified three phosphomolybdates. All the compounds were subjected to FTIR, TGA, SEM, EDS, XRD and XPS for physical characterization. Those compounds were mixed with some additives and casted on a GCE to fabricate modified GCE for electrochemical characterization by CV, GCD and EIS. All the compounds showed satisfactory results as pseudocapacitor.

## Experimental

### Chemicals and instrumentation

Ammonium heptamolybdate tetrahydrate, (NH_4_)_6_Mo_7_O_24_.4H_2_O were purchased from Sisco Research Laboratories Pvt. Ltd., India. Phosphoric acid, H_3_PO_4_, were purchased from RCI Labscan Ltd., Thailand. Manganese acetate, (CH_3_COO)_2_Mn.4H_2_O, was purchased from Sigma-Aldrich (USA). Imidazole, C_3_H_4_N_2_, were purchased from Merck, Life Science Pvt. Ltd., India. Sodium hydroxide, NaOH, were purchased from Loba Chemie, India. Sulphuric acid, H_2_SO_4_, were purchased from RCI Labscan Ltd., Thailand. Polyvinylidene fluoride (PVDF), carbon black (C-black) and N-methyl pyrrolidone, were purchased from TOB, Taiwan, China. Glassy carbon electrode (GCE), (diameter- 3 mm), reference electrode AgǀAgCl and counter electrode, Pt coil, were purchased from Metroholm, UAE. All of the work was done using deionized (DI) water.

To identify the functional groups in the prepared products, FTIR experiment was done using FTIR spectrometer (IR Tracer-100, Shimadzu Corporation, Japan). The structure and surface morphology and elemental mapping of prepared products were analyzed by SEM machine (JSM-7610F, Japan). A small amount of sample was taken for gold coating then analyzed by FESEM machine. Powder X-ray diffractometer (XRD: Bruker, D2PHASER) was used to analyze X-ray diffraction of prepared products with CU of Kα radiation at 2θ from 10° to 80°. The elemental composition of prepared products and the average valency state of Mn were analyzed by X-ray photoelectron spectroscopy (XPS, Al Kα; Axis Supra + , Kratos, UK). The thermal stability of prepared products were investigated using TGA machine (TGA-50, Shimadzu, Japan), from room temperature to 800 °C (10 °C/min, 2mL/min Air, Pt pan). CV, EIS and BCD experiments were carried out by using potentiostat (BioLogic, SP-300, France).

### Synthesis of Mn-modified phosphomolybdates

**Fig 1:**
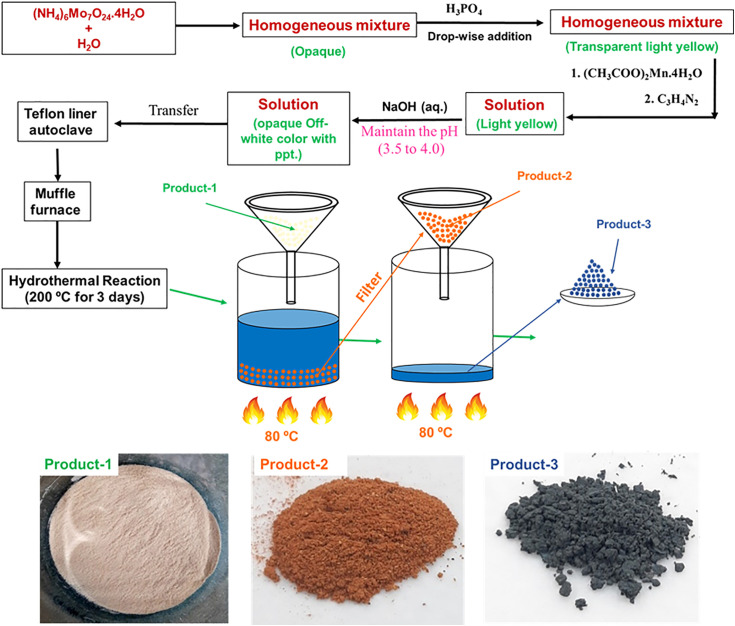
Synthetic route of Mn-modified phosphomolybdates.


1.0 mole of H_2_O was taken in a beaker and 0.6 M of ammonium heptamolybdate tetrahydrate, (NH_4_)_6_Mo_7_O_24_.4H_2_O, was added in it and mixed well to have a homogeneous solution. 15 M of H_3_PO_4_ was drop wise added into the solution and it turned from cloud like opaque solution to transparent light yellow. 3.0 M of manganese acetate, (CH_3_COO)_2_Mn.4H_2_O, followed by 2.4 M of imidazole, C_3_H_4_N_2_, was added in it and mixed well. Then 6.0 M of NaOH (aq.) was added in the solution to maintain the pH (3.5 to 4.0). Due to the addition of NaOH, off white colored ppt. was formed and the solution turned to opaque off white color. After that the solution was transferred to Teflon liner autoclave which was then kept in a muffle furnace at 200°C for 3 days [10]. Then the autoclave was cooled to room temperature and the solution was filtered followed by air drying to have the 1^st^ product (Product-1). Product-1 was off white colored solid power type substance. The remaining liquid filtrate was deep blue in color, which was heated at 80°C to remove the water content. While heating orange colored ppt. was formed and sedimented at the bottom of the beaker which was successively taken and filtrated. The heating was continued for 12 h until all the ppt. was formed and filtrated to have the 2^nd^ product (Product-2), which was orange colored solid powder. The remaining filtrated was again heated until all the water content was removed and dark blue colored solid powder like substance was left, which was the 3^rd^ product (Product-3). Unlike Product-1 and Product-2, Product-3 showed a sticky like property under the influence of heat and slight solubility in water. In addition, instead of a pure byproduct, Product-3 was considered to be the combination of several byproducts, or fragments of electrolytes, as the result of main reaction. So, when the filtrate was near to water content free, the heating was stopped and the filtrate was air dried to have Product-3 powder. Equation (1) could be the possible reaction for product formation and Fig 1 represents the synthetic route with the real pictures of all products.

$$\begin{array}{cc} & {{(NH}_{\text{4}})}_{\text{6}}{Mo}_{\text{7}}O_{\text{2}\text{4}}.{\text{4}H}_{\text{2}}O+\;H_{\text{2}}O+\;H_{\text{3}}PO_{\text{4}}+\;{\left({CH}_{\text{3}}Coo\right)}_{\text{2}}Mn.\text{4}H_{\text{2}}O+\;C_{\text{3}}H_{\text{4}}N_{\text{2}}\\ & +NaOH\to Product-\text{1}\;(off\;white)+Product-\text{2}\;(orange)+Product-\text{3}\;(dark\;blue) \end{array}$$ (1)

### Electrode preparation

Here, working electrode is GCE and active material is synthesized products (Product-1/Product-2/Product-3). Before every experiment, GCE was cleaned using ethanol:water (50%:50% v/v) solution in an ultrasonic bath for 30 min followed by polishing the electrode tip on a polishing pad containing alumina slurry. C-black was used to increase the conductivity of Product-1/Product-2/Product-3, PVDF was used as gluing agent and NMP as solvent; together PVDF and NMP was used as the binding agent. Product-1/Product-2/Product-3 (50%), C-black (40%) and PVDF (10%) were mixed well followed by the addition of NMP to have a viscous smooth slurry. Then the slurry was applied on the tip of a GCE to have a thin film on it. After that the modified GCE was dried at 70 °C for 30 min to have the final working electrode (Product-1@GCE/Product-2@GCE/Product-3@GCE). For electrochemical cell AgǀAgCl was used as reference electrode, Pt coil as counter electrode, and 0.5 M H_2_SO_4_ as electrolyte [10,29]. About 10 modified GCE of each synthesized products were fabricated for electrolytic testing.

## Results and discussion

**Fig 2:**
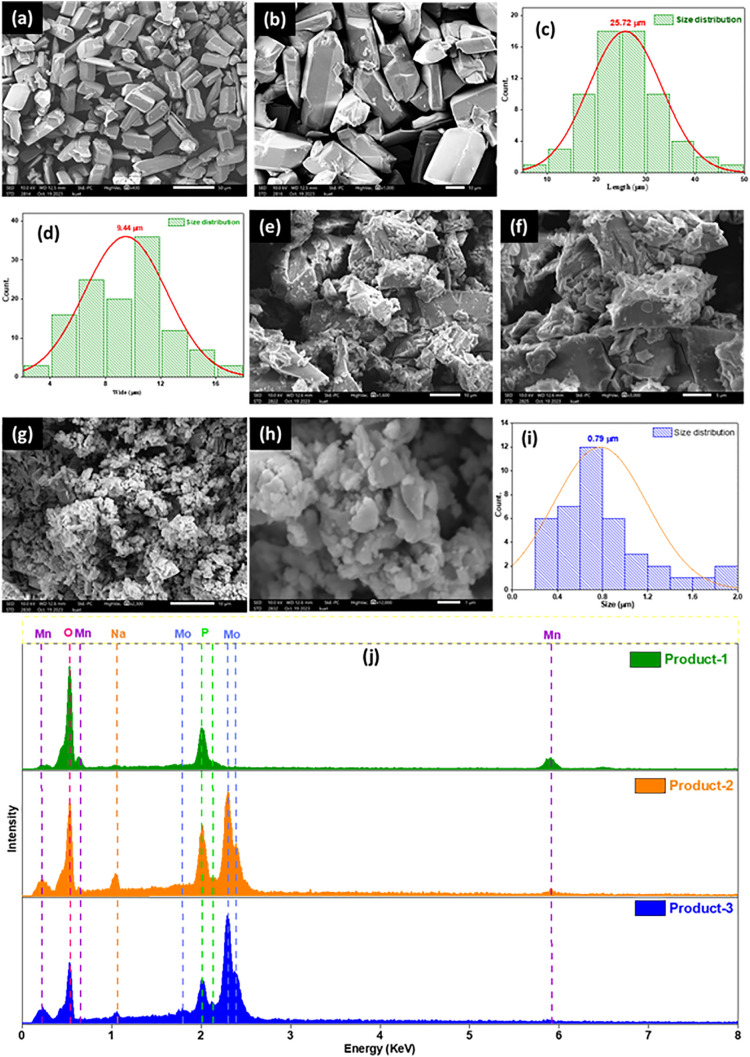
SEM images: Product-1 at scaling (a) 50 μm, (b) 10 μm and histogram according to the particles (c) length and (d) wide; Product-2 at scaling (e) 10 μm, (f) 5 μm, and corresponding histogram; Product-3 at scaling (g) 10 μm, (h) 5 μm; (j) EDS of all Products.

Fig 2 shows the surface morphology of prepared products. Product-1 had similar type of grains spread all over the surface (Fig 2(a,b)). The grains looked like small bricks having an average length of 25.72 μm and wide of 9.44 μm. These values were calculated using histograms (Fig 2(c,d)). The histogram of grain’s length (Fig 2(c)), was symmetrically unimodal in shape, which was different than unsymmetrical unimodal shape of histogram used for grain’s wide, which explained the Product-1 having grains of almost similar type lengths but different in wide. Besides some broken parts of brick like grains were also seen, which might arose during transfer of the product. Similar type of product [{Ag_5_(pz)_6_(H_2_O)_0.5_Cl}{PMo_11_MoO_40_}] was synthesized by Xingzhi, *et al.*, where pyrazine (pz) was used as ligand and Ag as metal instead of imidazole and Mn [10]. The surface of Product-2 (Fig 2(e,f)) looked like large particles covered with small particles of different sizes. It was difficult to calculate the grain size because if one side of the particle was noted, another side was not clear enough to note. While analyzing the surface of Product-2 (Fig 2(g,h)), histogram (Fig 2(i)) indicated an average grain size of 0.79 μm. The grains of Product-3 were very random in sizes and shapes, which was difficult to analyze. It was also noticed that the upper portion of all Product-2 grains were covered by similar like grains of Product-3. Since Product-2 was synthesized by heating Product-3 containing dark blue filtrate (after filtration of Product-1), Product-2 could be the aggregated product of Product-3. Because Product-3 had water affinity but Product-2 had not, Product-2 might be the polymer or a derivative of Product-3. Fig 2(j) represents the combined EDS spectrum of all prepared products. The EDX lines observed at 2.35, 0.51, and 1.98 keV were associated for K lines of the Mo, O, and P, respectively. Besides Mn showed K lining at 5.99, 6.57, and 0.577 keV. All the products (Product-1, 2 and 3) consisted the percentage of Mn of 31.72, 5.60, and 2.96, O of 52.07, 64.61, and 63.98, and P of 15.37, 12.12, and 8.51, respectively. Another important element was Mo which amount was 17.67 and 24.55% in Product-2 and Product-3 but feebly detectable for Product-1. For large size of crystals if Mo was used for kegging type polymer formation, the amount of it had to be very small and the position of it would be at the center of kegging monomer unit. Thus it might not be possible to detect Mo by EDS technique.

**Fig 3:**
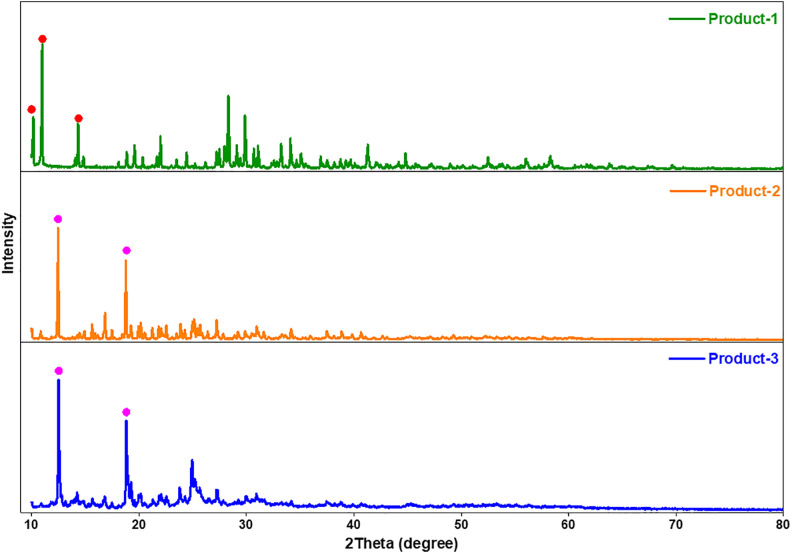
XRDs of prepared products.


Fig 3 shows the powder XRD of all products which indicated a lot of peaks at different 2θ position. There was no similarity of peaks of Product-1 with other two compounds, but Product-2 showed some similarities with Product-3. The XRD pattern of Product-1 looked similar as the kegging complexes explored by Xingzhi, *et al.* [10], first three peaks at 2θ = 10.16, 10.96 and 14.32° could be the planes responsible for Mo-O-Mo or O-Mo-O bonding in a kegging complex. Two peaks at 2θ = 12.45 and 18.79 of Product-2 were similar with two peaks of Product-3 at 2θ = 12.46 and 18.80, their peak intensities were also identical which could be distinguishable by bare eyes. After Product-1 was filtered, the dark blue colored filtrated was heated to synthesize Product-2, and after Product-2 was filtered Product-3 was collected after the sedimentation of remaining dark blue colored filtrate. So, it could be possible that there happened a polymerization reaction between Product-3 particles under influence of heat and Product-2 might be the polymer of Product-3 particles (monomers). Apart from those two distinguishable similar peaks, there were a lot of peaks with comparably lower intensities, whose peak positions were comparable but intensities were quite different. It might be said that during polymerization of Product-3 monomer new Product-2 polymer crystal was formed which unit cell molecular arrangement was a bit different from the unit cell of Product-3. The average crystal size of the prepared products were calculated using Scherer equation (2),

$$D=\frac{K\lambda }{\beta Cos\theta }$$ (2)

where, D = crystallite size in nm, K = Scherer constant, λ = wavelength of X-ray in nm, β = full wave half maximum (FWHM) of the XRD peaks and θ is Braggs angle (in radians) [30]. An average crystallite size of 125.27, 145.12, and 86.90 nm was observed for Product-1, 2 and 3, respectively. The crystallinity was calculated using following equation (3),


$$Crystallinity=\frac{Area\;of\;crystalline\;peaks}{Area\;of\;all\;peaks\;(Crystalline+Amorphous)}\times \text{1}\text{0}\text{0}\;\%$$ (3)

The crystallinity was found to be 63.13, 59.16, and 62.07% for Product-1, 2 and 3, respectively. We can say that all three compounds were highly crystalline in nature. The crystallite size of Product-3 was much smaller than that of Product-2 which could be the reason for being Product-3 as monomer and Product-2 as polymer.

**Fig 4:**
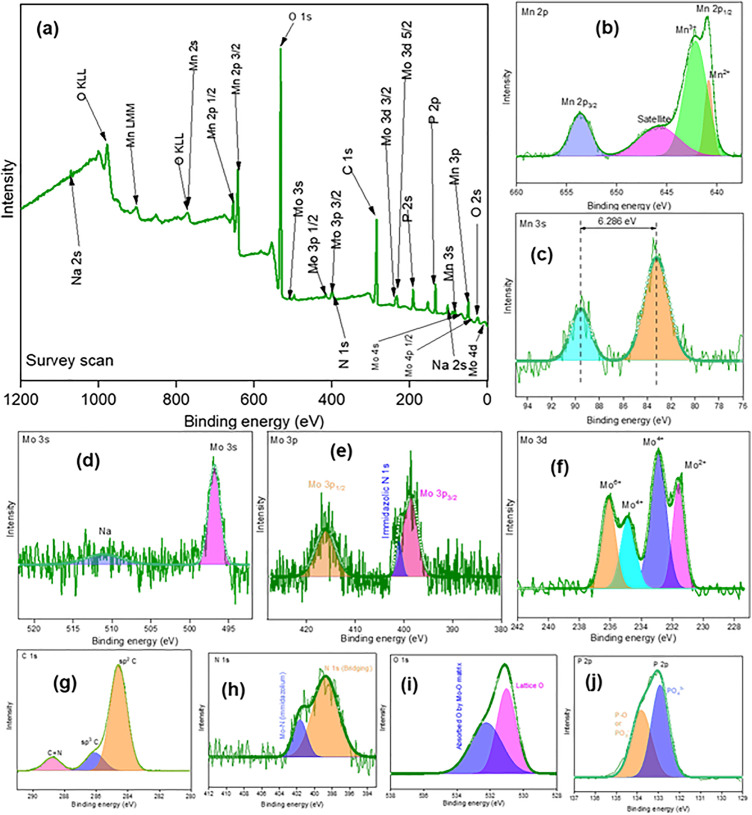
XPS spectrum of Product-1; (a) survey scan; Narrow spectrum of (b) Mn 2p, (c) Mn 3s, (d) Mo 3s, (e) Mo 3p, (f) Mo 3d, (g) C 1s, (h) N 1s, (i) O 1s, and (j) P 2p.

**Fig 5:**
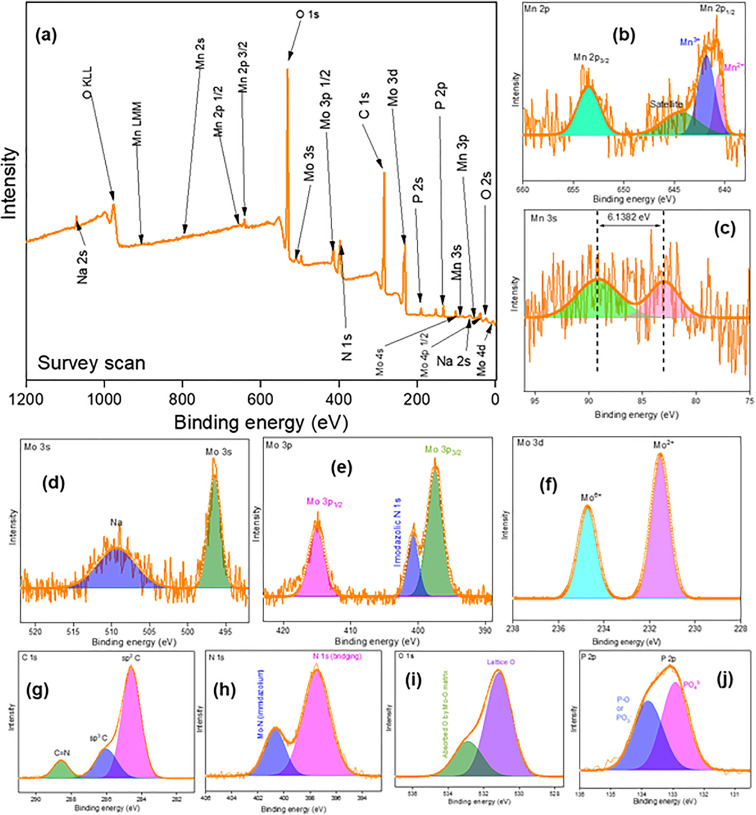
XPS spectrum of Product-2; (a) survey scan; Narrow spectrum of (b) Mn 2p, (c) Mn 3s, (d) Mo 3s, (e) Mo 3p, (f) Mo 3d, (g) C 1s, (h) N 1s, (i) O 1s, and (j) P 2p.

To understand the chemical composition of prepared products XPS was carried out. Fig 4(a),5(a),6(a) show the XPS survey scans of prepared Product-1, 2 and 3 within a binding energy range of +1200 to −2 eV. For all the products, presence of Mo, Mn, C, O, P, N and tiny amount of Na was confirmed. According to the no. of atoms, the percentages of Mo, Mn, C, O, P, N and Na were 3.91, 23.30, 13.54, 47.83, 7.9, 2.30, and 1.15%, 20.10, 2.39, 22.98, 41.53, 3.53, 6.83, and 2.6%, and 15.51, 1.06, 31.29, 43.56, 1.84, 5.58, and 0.58%, in Product-1, 2, and 3, respectively. For, Product-1, narrow spectrum of Mn 2p (Fig 4(b)), showed the presence of Mn^2+^ (640.83 eV) and Mn^3+^ (642.14 and 645.95 eV). Mn 3s narrow spectrum (Fig 4(c)) showed two peaks at 83.25 and 89.55 eV having a separation of 6.286 eV. The average valency of Mn was calculated according to the conventional linear equation (4),

**Fig 6:**
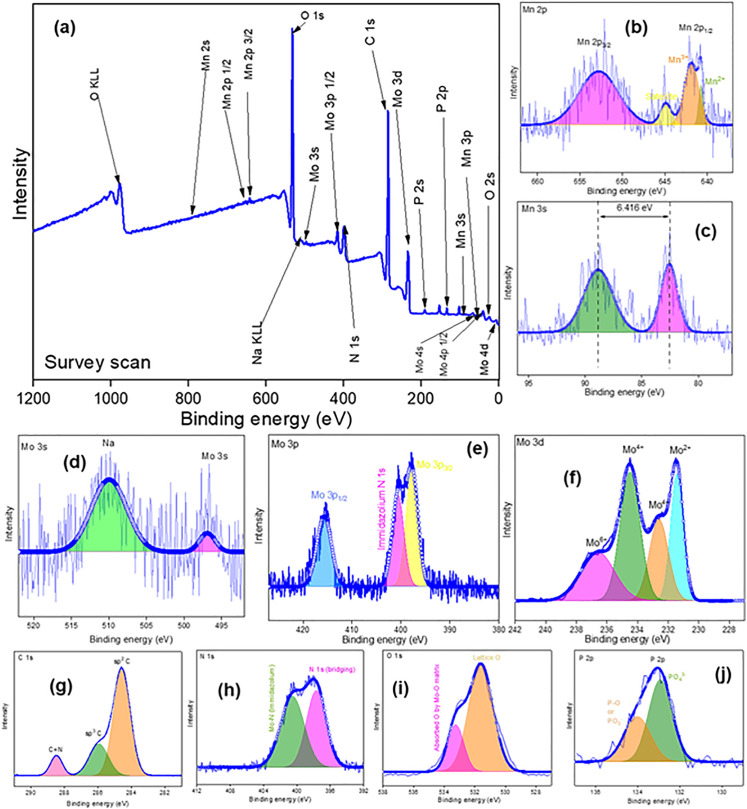
XPS spectrum of Product-3; (a) survey scan; Narrow spectrum of (b) Mn 2p, (c) Mn 3s, (d) Mo 3s, (e) Mo 3p, (f) Mo 3d, (g) C 1s, (h) N 1s, (i) O 1s, and (j) P 2p.


$$V_{Mn}=\text{7}.\text{8}\text{7}\text{5}-\text{0}.\text{8}\text{9}\text{3}\;\Delta E_{\text{3}s}$$ (4)

The calculated values of average valency of Mn was 2.26, indicated Mn has both +2 and +3 oxidation state in Product-1 [31]. Narrow spectrum of Mo 3s, 3p, and 3d (Fig 4(d, e, f)) denoted the presence of Mo having +2, + 4, and +6, at peak positions 231.58, 232.90 and 234.89, and 236.11 eV [32]. Bonding of Mo with imidazolic N 1s was confirmed at 400.92 eV [33]. Very slight wide satellite peak was observed at 511.08 eV for Na. While washing Product-1 some of Na might remain in it. Narrow spectrum of C 1s (Fig 4(g)) showed the presence of sp^2^ C and sp^3^ C at 284.59 and 286.11 eV, and C = N bond at 288.77 eV could be responsible for imidazolium unit [34]. N 1s narrow spectrum (Fig 4(h)) indicated N at 398.75 eV as bridging element in imidazolium unit and at 401.63 eV Mo-N (imidazolium) bond for N in Mo-kegging complex [35]. O 1s spectrum (Fig 4(i)) designated Lattice O at 531.00 eV and oxygen absorption by Mo-O matrix at 532.24 eV [36]. P 2p spectrum (Fig 4(i)) suggested the existence of P-O at 133.80 eV and PO_4_^3-^ at 132.92 eV [37]. Similar narrow spectrums of Mo, Mn, C, N, O, and P were done for Product-2 (Fig 5(b-j)) and Product-3 (Fig 6(b-j)). For Product-2, existence of both Mn^2+^ (640.60 eV) and Mn^3+^ (641.83 eV) was noticed. In Mn 3s, a doublet was seen, and average valency of Mn was found to be 2.39. Presence of Mo^2+^ and Mo^4+^/Mo^6+^ was indicated at 231.52 and 234.75 eV. Bonding of Mo with imidazolic N was at 400.76 eV, small amount of Na was present at 509.41 eV, might remained while washing the Product-2. C having sp^2^ and sp^3^ was at 284.57 and 286.03 eV, C = N in imidazolium unit was at position 288.54 eV. Bridging N in imidazolic unit was at 397.48 eV and bonding of Mo with imidazolic N was at 400.66 eV. Lattice O was denoted at 531.18 eV and absorption of O by Mo-O matrix was located at 532.91 eV. Existence of P-O bond and P in PO_4_^3-^ was located at 133.81 and 132.92 eV. For Product-3, Mn had both 2+ and 3 + oxidation states (Fig 6(b)), and an average valency was found to be 2.14 (Fig 6(c)). Mo^6+^ was present along with Mo^4+^ and Mo^2+^ (Fig 6(f)). Imidazolic N 1s was also seen (Fig 6(e)). Since after Product-2 was collected all the filtrate was heated for water removal and concentrated to have Product-3, it will also contained possibly unreacted NaOH or other compounds. Thus appreciable amount of Na was noticed (Fig 6(d)). C 1s spectrum (Fig 6(g)) was similar as Product-2, sp^2^ C and sp^3^ C were present along with C = N. Also Mo-N (imidazolic) was noticed (Fig 6(h)). Besides, Mo-O (Fig 6(i)) and P-O (Fig 6(j)) bonds were also located.

**Fig 7:**
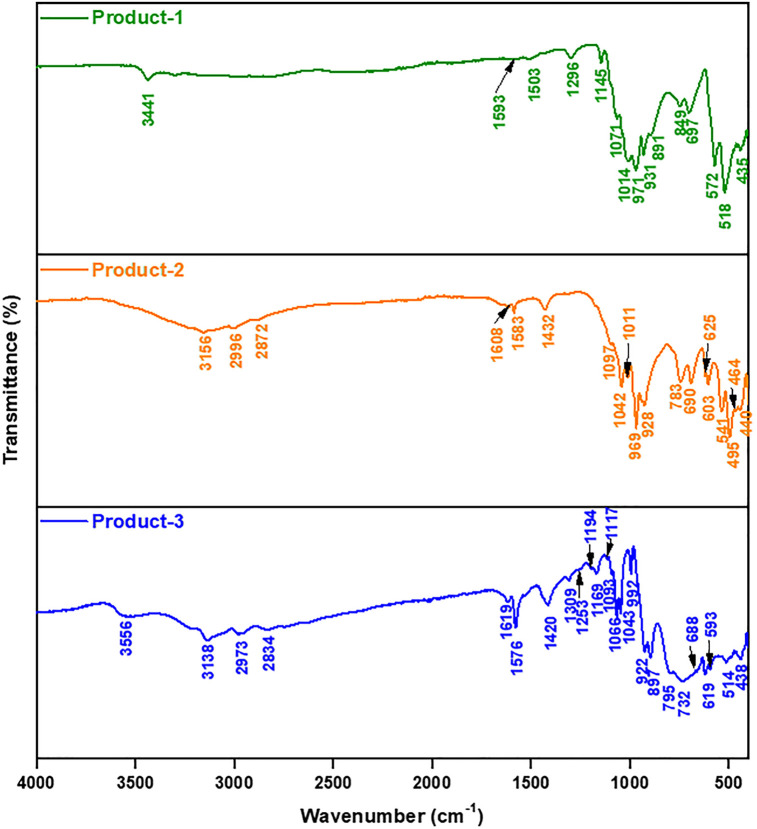
FTIR spectra of prepared products.

Fig 7 shows the FTIR spectra of prepared products and the analyses were done within the range of 400–4000 cm^-1^. Peaks at 3441 (Product-1), 3156 (Product-2), 3556 (Product-3) and 3138 (Product-3) cm^-1^ indicated the stretching vibration [10], and peaks at 1593 (Product-1), 1608 (Product-2) and 1619 (Product-3) cm^-1^, indicated the bending vibration of O-H group, which were responsible for the moisture content in KBr plate. Peaks at 2996 (Product-2), 2872 (Product-2), 2973 (Product-3) and 2834 (Product-3) cm^-1^ showed the stretching vibration of C-H bond [38], and peaks at 1583 (Product-2) and 1576 (Product-3) cm^-1^ directed the C = C bonding [39], which arose due to the presence of imidazolic unit in the structures. Peaks at 1503 (Product-1) cm^-1^ was for the stretching vibration [40], and peaks at 1432 (Product-2) and 1420 (Product-3) cm^-1^ were for the bending vibration of N-H groups [38] in immidazolic unit. Stretching vibration of C-N and bending vibration of C-H bond were located at 1296 (Product-1), 1253 (Product-3), 1309 (Product-3) cm^-1^ [40] and 1145 (Product-1), 1117 (Product-3), 1169 (Product-3), 1194 (Product-3) cm^-1^ [41], also responsible for imidazolic unit. Peaks at 1071 (Product-1), 1014 (Product-1), 1097 (Product-2), 1042 (Product-2), 1011 (Product-2), 1093 (Product-3), 1066 (Product-3), 1043 (Product-3) cm^-1^ were accountable for stretching vibration of P-O bond and proved the presence of P-O bond in PO_4_ tetrahedron [42]. Peaks at 971 (Product-1), 931 (Product-1), 891 (Product-1), 849 (Product-1), 969 (Product-2), 928 (Product-2), 992 (Product-3), 922 (Product-3), 897 (Product-3) cm^-1^ directed terminal Mo-O or Mo = O groups [17,42]; 697 (Product-1), 783 (Product-2), 690 (Product-2), 625 (Product-2), 795 (Product-3), 732 (Product-3), 688 (Product-3), 619 (Product-3) cm^-1^ indicated the presence of stretching vibration of Mo-O-Mo bridging or edge sharing of MoO_6_ octahedra [42,43]; 572 (Product-1), 518 (Product-1), 435 (Product-1), 541 (Product-2), 440 (Product-2), 464 (Product-2), 495 (Product-2), 603 (Product-2), 593 (Product-3), 514 (Product-3), 438 (Product-3) focused the existence of bending vibration of bridging symmetric and asymmetric O-Mo-O bonds [38,43]. Concisely, the FTIR spectra suggested the formation of phosphomolybdates which also are in good agreement with the XPS data.

**Fig 8:**
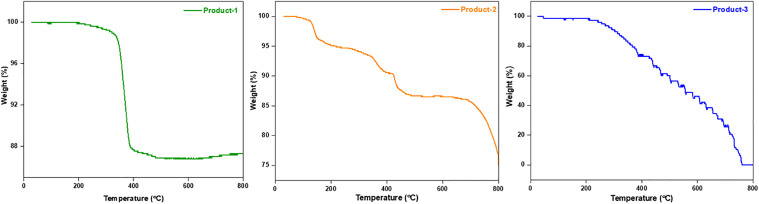
TGA of prepared products.

The thermal property of prepared products were checked by TGA technique (Fig 8). Product-1 seemed to be the most stable among all. Product-1 retained its identity up to 198 °C, then started to loss its weight slowly to 98% till 339 °C. Then a drastically weight loss were observed to 87% till 389 °C, then again slowly loosed its weight to 86% till 479 °C. Only 14% of its total weight was loosed till 800 °C which implied Product-1 as a strong polymer type material. Product-2 retained its identity up to 76 °C, the till 124, 152, 418, 490, 685, and 800 °C it loosed 99, 96, 90, 87, 86, and 75% weight of its initial value. Product-2 showed lesser stability as compared to Product-1 but has greater stability than Product-3. Product-3 retained its stability till 207 °C, but then it began to lose its weight gradually as the temperature rose and at 760 °C it completely loosed all of its weight. Unlike other two products, Product-3 might have internal heat sensitive weak bonds.

**Fig 9:**
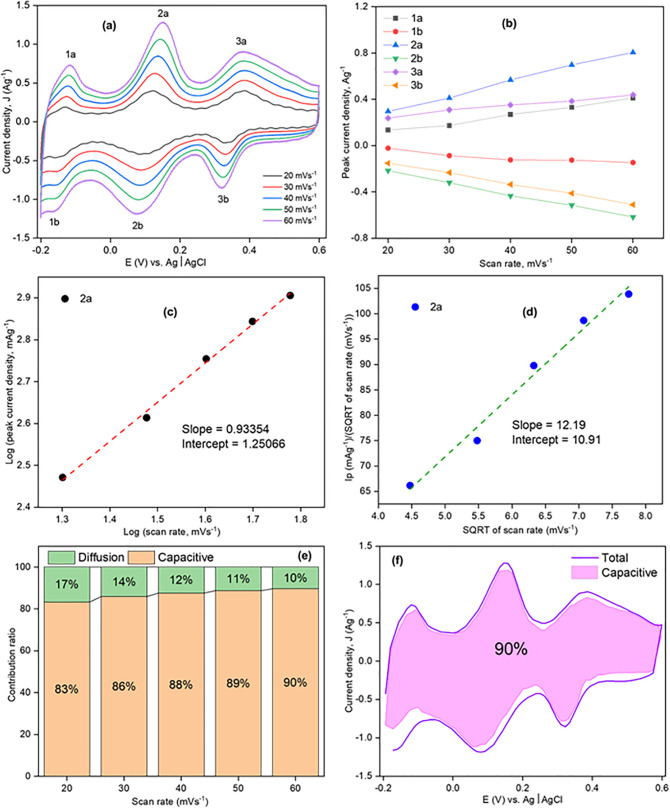
CV of Product-1@GCE (a) at different scan rates, (b) scan rate vs. peak current density, (c) Log (scan rate) vs. Log (peak current density), (d) SQRT (scan rate) vs. Ip/SQRT (scan rate), (e) contribution ratio at different scan rates, and (f) capacitive contribution coverage at 60 mVs^-1^.

**Fig 10:**
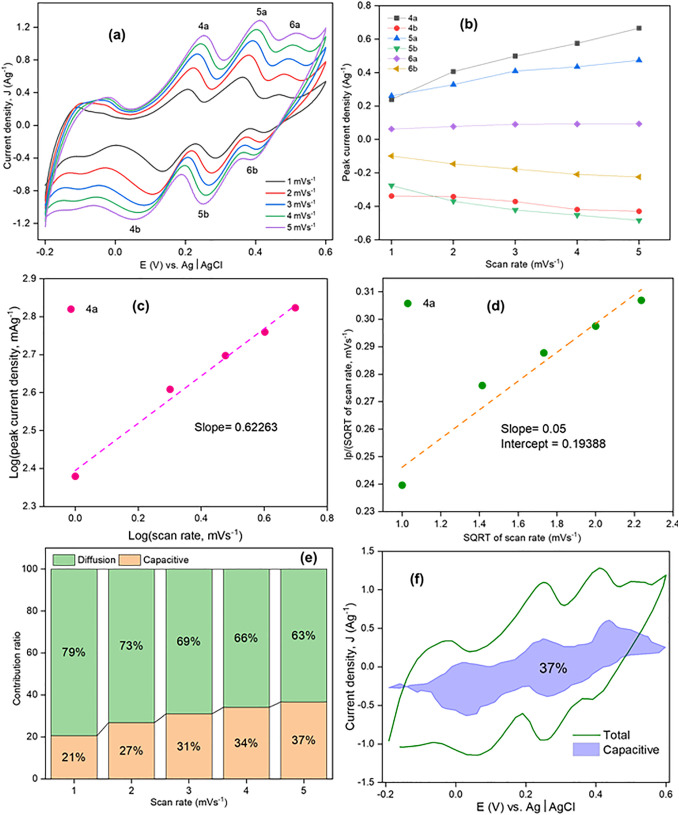
CV of Product-2@GCE (a) at different scan rates, (b) scan rate vs. peak current density, (c) Log (scan rate) vs. Log (peak current density), (d) SQRT (scan rate) vs. Ip/SQRT(scan rate), (e) contribution ratio at different scan rates, and (f) capacitive contribution coverage at 5 mVs^-1^.

**Fig 11:**
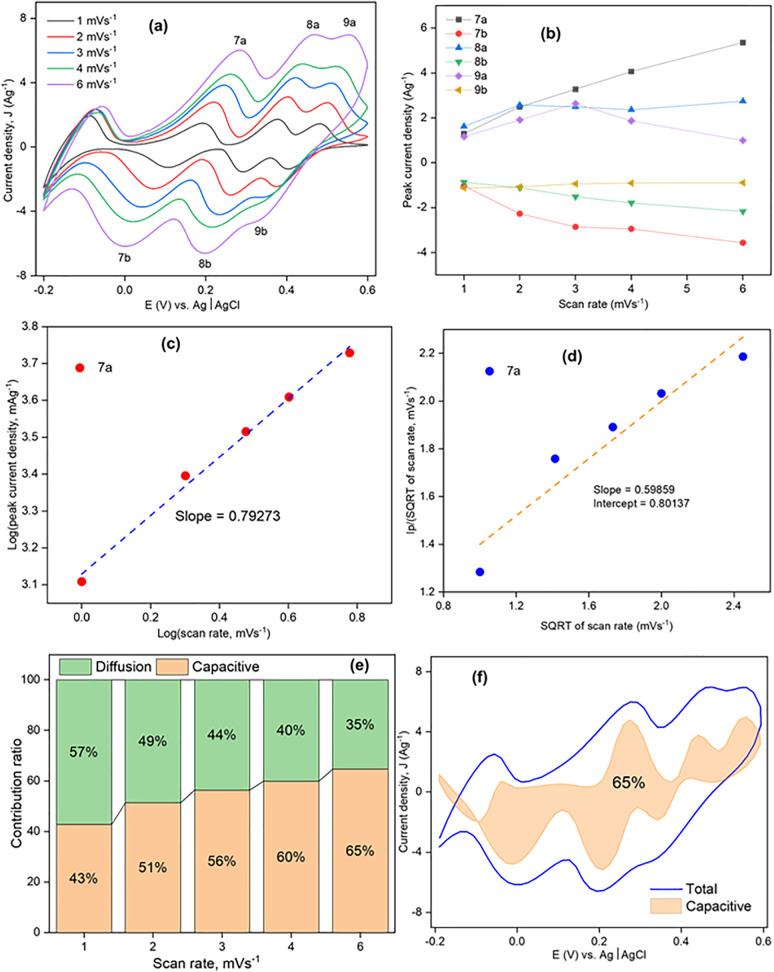
CV of Product-3@GCE (a) at different scan rates, (b) scan rate vs. peak current density, (c) Log (scan rate) vs. Log (peak current density), (d) SQRT (scan rate) vs. Ip/SQRT (scan rate), (e) contribution ratio at different scan rates, and (f) capacitive contribution coverage at 6 mVs^-1^.

To examine the electrochemical behavior of Product-1/2/3, CVs were taken by Product-1/2/3@GCE having 0.5 M of H_2_SO_4_ (aq.) solution. Fig 9–11 show the CVs of Product-1@GCE, Product-2@GCE, and Product-3@GCE. The electrochemical windows were kept from −0.2 to +0.6 V [10]. All electrodes showed three pairs of distinct redox peaks, which indicated Product-1, 2 and 3 as completely three different compounds. For Product-1@GCE, CVs were taken (Fig 9(a)) at different scan rates (20, 30, 40, 50, and 60 mVs^-1^). With the increasing of scan rates, the peaks heights increased, anodic peaks shifted slightly right and cathodic peaks towards left. From lowest to the highest scan rates designated peaks 1a, 2a, 3a, 1b, 2b, and 3b shifted from −0.1286, + 0.12003, + 0.38099, + 0.1397, + 0.0986, and +0.3336 V to −0.1172, + 0.1503, + 0.3821, −0.1623, + 0.0785, and 0.3202 V, respectively. For Product-2@GCE, CVs were taken (Fig 10(a)) at different scan rates (1, 2, 3, 4, and 5 mVs^-1^). Here, the scan rates are comparatively lower than those for Product-1@GCE, because higher scan rates cause broadening of peaks, sometimes peak vanished, which indicated slower electron transfer process. Like Product-1@GCE, it also showed increase of peaks current and shifting of peaks potential toward right for anodic and left for cathodic ones. From lowest to the highest scan rates designated peaks 4a, 5a, 6a, 4b, 5b, and 6b shifted from +0.2063, + 0.3633, + 0.4669, + 0.13794, + 0.28599, and +0.42369 V to +0.254, + 0.4112, + 0.51466, + 0.052, + 0.2494, and +0.3896 V, respectively. Like Product-2@GCE, Product-3@GCE also showed the increase of peaks current with the increase of scan rates to comparatively lower scan rates as compared to Product-1@GCE, and the shifting of peaks at 7a, 8a, 9a, 7b, 8b, and 9b were from +0.1925, + 0.3758, + 0.4828, + 0.10826, + 0.2926, and +0.40435 V to +0.28369, + 0.4635, + 0.5535, + 0.0009, + 0.19712, and +0.33023 V, respectively at scan rates 1, 2, 3, 4, and 6 mVs^-1^ respectively.

The behavior of the CVs was tested by Dunn’s method eqn (5).


Log i=Log a+b Log v (5)

Where, a and b (slope) are constant. The value of b is between 0.5 and 1.0. If the value of b is 0.5, it denotes a diffusion- controlled behavior (Q_d_) and if it is 1.0, it implies capacitive process (Q_c_) [44]. Fig 9(b),10(b),11(b) show the corresponding graphs of scan rate vs. peak current density, which indicated almost linear relationship. Keeping peaks at 2a, 4a, and 7(a), Log(scan rate) vs. Log(peak current density) was drawn (Fig 9(c),10(c),11(c)), linear fitting of the data showed the value of slope was 0.9335, 0.62263, and 0.79273, respectively for Product-1@GCE, Product-2@GCE, and Product-3@GCE, indicated high capacitive contribution of the electrodes. Another graph (Fig 9(d),10(d),11(d)) were drawn between SQRT of scan rate and the fraction of peak current density and SQRT of scan rate, to evaluate the capacitive contribution, using following eqn (6),


$$i_{p}\;(\nu )=\;k_{\text{1}}\nu +k_{\text{2}}\nu^{\frac{\text{1}}{\text{2}}}$$

$$\text{Or},\frac{i_{p}}{v^{\frac{\text{1}}{\text{2}}}}=\;k_{\text{1}}v^{\frac{\text{1}}{\text{2}}}+k_{\text{2}}$$ (6)

Where, k_1_ and k_2_ are the slope and intercept of $v^{\frac{\text{1}}{\text{2}}}\;$ vs $\frac{i_{p}}{v^{\frac{\text{1}}{\text{2}}}}$ plot. The contribution from pseudo-charge storage is k_1_ν, and the insertion type capacity is k_2_*v*^1/2^ [45]. From the graphs slope (k_1_) and intercept (k_2_) were 12.19 and 10.91, 0.05 and 0.19388, 0.59859 and 0.80137, for Product-1@GCE, Product-2@GCE, and Product-3@GCE, respectively. The calculated values of diffusion and capacitive contribution ratio for Product-1@GCE (Fig 9(e)) were 17, 14, 12, 11, and 10% and 83, 86, 88, 89, and 90%, respectively, at scan rates 20, 30, 40, 50, 60 mVs^-1^, respectively. Similar calculations were done for other two electrodes too. The calculated values of diffusion and capacitive contribution ratio of Product-2@GCE and Product-3@GCE were 79, 73, 69, 66 and 63% and 21, 27, 31, 34, and 37%; 57, 49, 44, 40, and 35% and 43, 51, 56, 60, and 65%, respectively, at scan rates 1, 2, 3, 4, and 5 mVs^-1^ and 1, 2, 3, 4, and 6 mVs^-1^, respectively. In all cases capacitive contribution increased with scan rates. The highest capacitive contribution coverage of Product-1@GCE, Product-2@GCE, and Product-3@GCE is shown in Fig 9(f),10(f),11(f) with respect to the total contribution.

**Fig 12:**
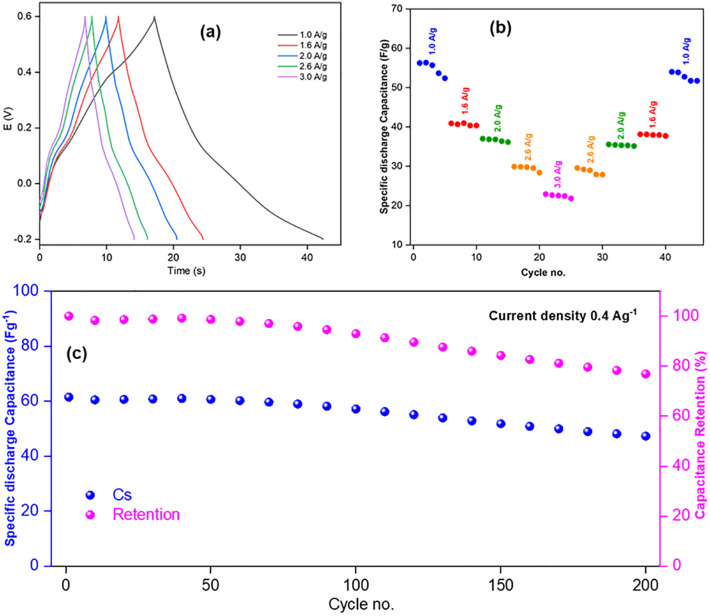
GCD of Product-1@GCE (a) at different current densities and their (b) rate capabilities, (c) cycling over 200 cycles at current density 0.4 Ag^-1^.

**Fig 13:**
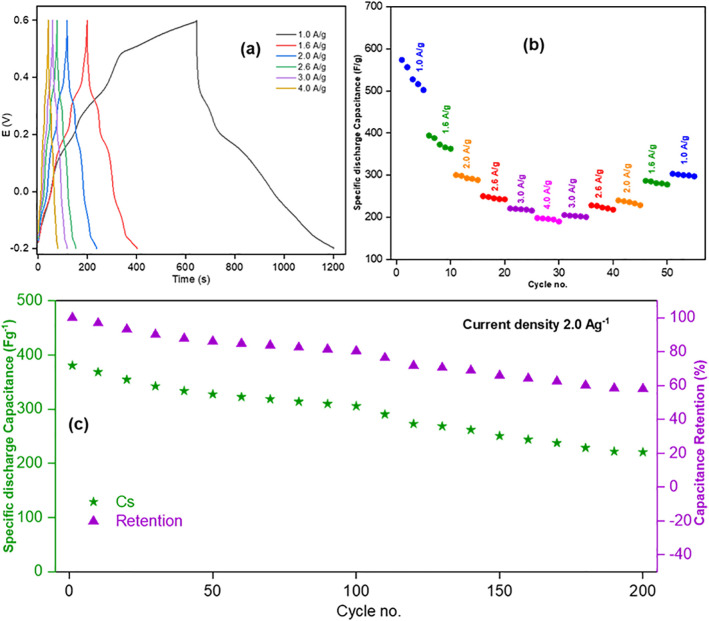
GCD of Product-2@GCE (a) at different current densities and their (b) rate capabilities, (c) cycling over 200 cycles at current density 2.0 Ag^-1^.

**Fig 14:**
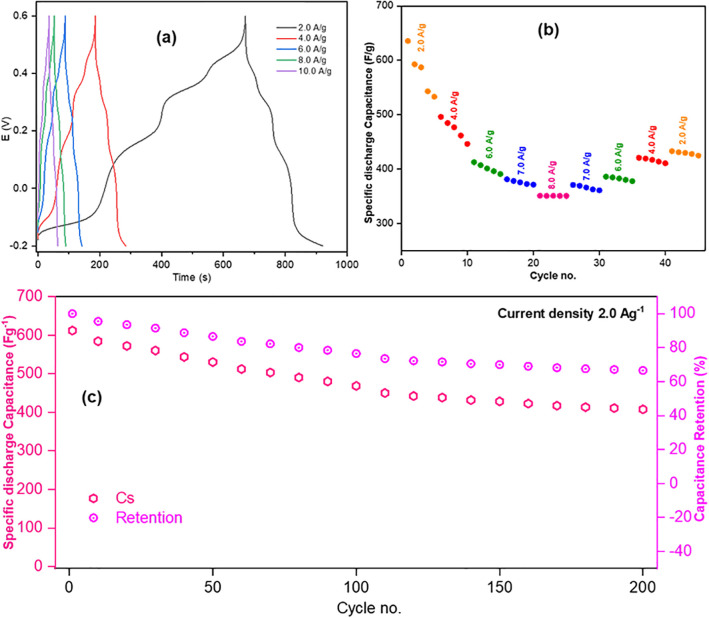
GCD of Product-3@GCE (a) at different current densities and their (b) rate capabilities, (c) cycling over 200 cycles at current density 2.0 Ag^-1^.

GCD of all three electrodes were studied within the voltage range −0.2 to +0.6 V to assess their capacitance properties [10]. GCD curves of the electrodes are shown in Fig 12(a), Fig 13(a), and Fig 14(a). For Product-1, 2 and 3@GCE, current densities of 1.0, 1.6, 2.0, 2.6, and 3.0 Ag^-1^, 1.0, 1.6, 2.0, 2.6, 3.0, and 4.0 Ag^-1^ and 2.0, 4.0, 6.0, 8.0, and 10.0 Ag^-1^ were applied, respectively, and the specific capacitances (C_s_) were decreased with the increasing of applied current densities [46]. Among these, Product-3@GCE showed highest specific capacitances as compared to others. The rate capabilities (Fig 12(b), 13(b), and 14(b)) were also assessed, for every applied current densities, five cycles were run from lower to higher current density and then higher to lower current density to analyze the retention of last data as compared to the first data. Product-1@GCE showed very satisfactory rate capability. At current densities 1.0, 1.6, 2.0, 2.6, 3.0, 2.6, 2.0, 1.6, and 1.0 Ag^-1^, the specific discharge capacitances were 56.25, 40.89, 37.01, 29.9, 22.897, 29.5625, 35.565, 38.115, and 53.995 Fg^-1^, respectively and it retained almost 95.99% of its initial value at 1.0 Ag^-1^. Product-2@GCE showed higher values of C_s_ as compared to Product-1@GCE, which is almost ten times higher than that of Product-1@GCE. At current densities 1.0, 1.6, 2.0, 2.6, 3.0, 4.0, 3.0, 2.6, 2.0, 1.6, and 1.0 Ag^-1^, the discharge C_s_ were 573.75, 394.12, 300.53, 250.25, 221.22, 198.43, 205.55, 228.564, 240.36, 286.864, and 303.519 Fg^-1^, respectively, and it retained almost 52.90% of its initial value at 1.0 Ag^-1^. The fading of discharge C_s_ is due to the formation of stable compounds during GCD. Similarly, for Product-3@GCE, at current densities 2.0, 4.0, 6.0, 7.0, 8.0, 7.0, 6.0, 4.0, and 2.0 Ag^-1^, the discharge C_s_ were 635.37, 495.85, 412.37, 380.93, 350.57, 370.475, 385.574, 420.574, and 432.5 Fg^-1^, respectively, and it retained almost 68.03% of discharge C_s_ of its initial values at 2.0 Ag^-1^. Product-3@GCE showed extremely higher C_s_ as compared to other two electrodes. This could be explained in terms of electron availability and the smaller particle size as compared to the others. Like Product-2@GCE, Product-3@GCE also showed fading of C_s_ from the beginning. Though the particle size was smaller, the water affinity character of Product-3 caused thinning of electrode surface, which was responsible for the drastically decrease of discharge C_s_ from the starting 5 cycles. Cycling of GCD were also done (Fig 12(c), 13(c), and 14(c)) for 1000 cycles at 0.4, 2.0, and 2.0 Ag^-1^ current densities for Product-1, 2 and 3@GCE, respectively. The retention of discharge C_s_ of Product-1, 2 and 3@GCE were 76.87, 58.00, and 66.57%, respectively, after 200 cycles. Though the discharge C_s_ of Product-1 and 2@GCE were higher than that of Product-1@GCE, the retention is much lower. These implied the flexible and stable internal structure of Product-1 for capacitive action over other two electrodes.

GCD cycling of 1000 cycles for all products were not equally satisfactory. The complete 1000 cycles of GCD has been shown in [S1, S2, S3 Fig]. The specific discharge capacity retention of Product-1, −2, and −3@GCE, with respect to the applied current density mentioned previously, after 100, 200, 300, 400, 500, 600, 700, 800, 900, and 1000 cycles were 92.92, 76.87, 67.16, 60.26, 55.24, 51.32, 48.19, 45.61, 43.44, and 41.68%; 80.33, 58.00, 36.14, 28.24, 25.03, 23.12, 15.21, 5.51, 5.42, and 5.06%; 76.50, 66.57, 54.98, 35.51, 20.66, 10.85, 2.37, 2.12, 1.82, and 1.46%, respectively. We can see that after 1000 cycles Product-2@GCE, and Product-3@GCE showed drastic capacitance loss to 5.06 and 1.46%, indicating similar behavior, also they indicated the irreversible chemical interaction of the electrolyte and the active materials leading surface product formation causing blockage of available reaction sites for pseudocapacitance performances.

Another GCD experiments were carried out (S4 Fig) keeping only carbon black leaving the active material to analyze how much capacitance arose from the carbon black. Electrode was prepared as the previous way keeping 90% of carbon black with 10% of PVDF with required amount of NMP as solvent. GCDs were carried out by applying current densities 0.4 Ag^-1^ and 2.0 Ag^-1^ for 1000 cycles. At 0.4 Ag^-1^ it showed initial specific discharge capacitance of 8.8 × 10^−2^ Fg^-1^, which after 1000 cycles decreased to 7.8 × 10^−2^ Fg^-1^. At 2.0 Ag^-1^ it showed initial specific discharge capacitance of 2.3 × 10^−2^ Fg^-1^, which after 1000 cycles decreased to 2.2 × 10^−2^ Fg^-1^. We can see that the Carbon black used in our experiment is only conducting material but having no appreciable amount of capacitance as compared to the active materials.

**Fig 15:**
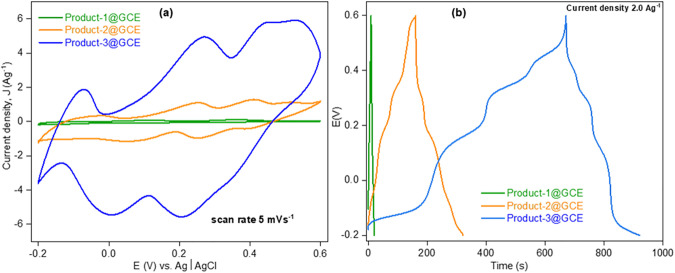
Combined (a) CVs and (b) GCD curves of all electrodes at scan rate 5 mVs^-1^ and current density 2.0 Ag^-1^, respectively.

The CVs and GCDs of all three electrodes were compared (Fig 15(a,b)). By comparing the three CVs at same scan rate of 5 mVs^-1^, it was found that the current density of Product-3@GCE were extremely higher than that of others, which is almost 49 and 10 times higher than Product-1 and 2@GCE, respectively. For GCD, The discharge C_s_ were also higher (635.37 Fg^-1^) than others (37.01 and 380.45 Fg^-1^), which is almost 17 and 10 times higher than Product-1 and 2@GCE, respectively. Besides, Product-3@GCE took almost 45 and 3 times greater times to complete first GCD cycle as compared to Product-1 and 2@GCE, respectively. A list of different POM-based electrode materials and their specific discharge capacitances with respect to applied current densities are given in Table 1. Product-1, 2 and 3 showed satisfactory results as compared with other products.

**Table 1:** List of POM-based electrode materials and their specific discharge capacitances with respect to applied current densities.

Electrode materials	Specific discharge capacitance (Fg^-1^)	Applied current density (Ag^-1^)	References
$\left\{\text{Cu}(\text{pra}{)}_{\text{2}}\}[{\left\{\text{Cu}(\text{pra}{)}_{\text{2}}\right\}}_{\text{3}}\left\{\text{P}\overset{VI}{\underset{\text{11}}{\text{Mo}}}{\text{Mo}}^{VI}O_{\text{4}\text{0}}\right\}\right]$	672.2	2.4	[47]
[Cu^I^H_2_(C_12_H_12_N_6_)(PMo_12_O_40_)]·[(C_6_H_15_N)(H_2_O)_2_]	249.0	3.0	[27]
[Cu^II^_2_(C_12_H_12_N_6_)_4_(PMo^VI^_9_Mo^V^_3_O_39_)]	154.5	3.0	[27]
$\left[\overset{I}{\underset{\text{4}}{Cu}}H_{\text{2}}{\left(btx\right)}_{\text{5}}{\left(P{Mo}_{\text{1}\text{2}}O_{\text{4}\text{0}}\right)}_{\text{2}}\right].{\text{2}H}_{\text{2}}O$	237.0	2.0	[28]
RGOs/PILs	408	0.5	[48]
SWCNT-TBA-PV_2_Mo_10_	444	2.0	[49]
PMo_12_/PPy	300	5.0	[50]
PolyIndole/PV_2_Mo_10_	198.09	0.2	[51]
[Cu^I^_6_(C_12_H_12_N_6_)_6_(PW^VI^_9_WV_3_O_40_)]2H_2_O	62.9	2.0	[28]
[Cu^I^_4_H_2_(C_12_H_12_N_6_)_5_(PMo_12_O_40_)_2_]2H_2_O	220.6	2.0	[28]
[Cu^II^Cu^I^_3_(C_12_H_12_N_6_)_5_(SiMo^VI^_11_MoVO_40_)]4H_2_O	135.7	2.0	[28]
[Cu^II^_2_(C_12_H_12_N_6_)_4_(PMo^VI^_9_Mo^V^_3_O_39_)]	146.7	2.0	[27]
[Cu^I^H_2_(C_12_H_12_N_6_)(PMo_12_O_40_)](C_6_H_15_N) [(H_2_O)_2_]	239.2	2.0	[27]
[Ag_5_(C_2_H_2_N_3_)_6_][H_5_SiW_12_O_40_]	29.8	0.5	[27]
[Ag_5_(C_2_H_2_N_3_)_6_][H_5_SiMo_12_O_40_]	155.0	0.5	[52]
[Ag_5_(C_2_H_2_N_3_)_6_][H_5_SiMo_12_O_40_]@15%GO	230.2	0.5	[52]
[H(C_10_H_10_N_2_)Cu_2_][PW_12_O_40_]	106.4	2.0	[53]
[H(C_10_H_10_N_2_)Cu_2_][PMo_12_O_40_]	232	2.0	[53]
Product-1	37.01	2.0	This work
Product-2	380.45	2.0	This work
Product-3	635.37	2.0	This work

**Fig 16:**
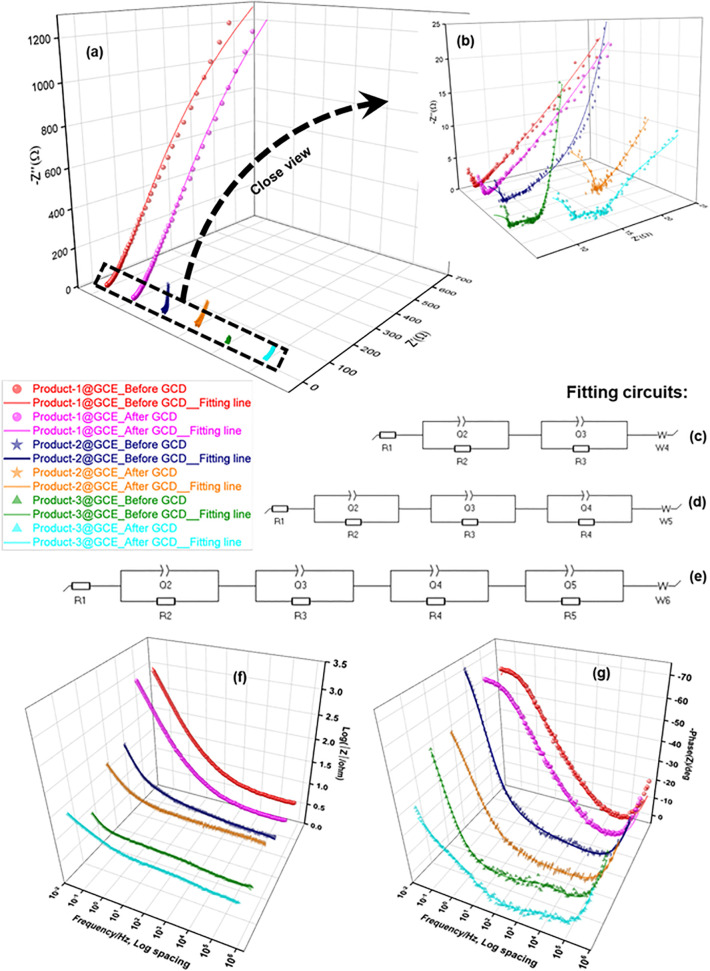
EIS of Modified Product-1, 2 and 3@GCE during GCD testing; (a) Nyquist plot and (b) its close view at high frequency range; (c, d, e) fitting circuits; Bode plots of frequency dependent (f) impedance and (g) phase angle.

Fig 16 shows the EIS profile of fabricated Product-1, 2 and 3@GCE. This experiment was carried out at applied potential +0.5 V within the frequency range 1 MHz to 100 mHz. This value of applied potential is higher than the potential of oxidation peaks of all electrodes obtained by CV experiments. EIS were recorded for both before and after 200 cycling of GCD. Three sets of Nyquist plots (Fig 16(a),(b)) were found and were analyzed by three different circuits. For Product-1, 2 and 3@GCE, equivalent circuits showing in Fig 16(c)-(e) were used, respectively, for fitting data evaluation [54]. Circuit (c) is R1 + Q2/R2 + Q3/R3 + W4, (d) is R1 + Q2/R2 + Q3/R3 + Q4/R4 + W5, and (e) is R1 + Q2/R2 + Q3/R3 + Q4/R4 + Q5/Q5 + W6; where, R = resistance, Q = non-ideal capacitance induced by non-uniform or porous characteristics, typically represented by constant phase elements (CPE), and W = Warburg impedance or resistance to mass transfer in bulk layer at low frequencies. R1 is valid for the resistance of solution in the three electrode system (R_s_), Q2 is responsible for the double layer capacitance (Q_dl_), R2 indicates electrode resistance (R_f_) due to solution electrolyte interface (SEI), Q3 and R3 is for the charge transfer capacitance (Q_ct_) and resistance (R_ct_), Q4, Q5, R4, R5 arose due to the formation of secondary/tertiary capacitors loosely held with the electrode within the cell at middle frequency region. Because of these self-made capacitors, mass transport system hampered resulting slower transfer of electrons [55]. In case of Product-1@GCE, at high frequency region the Nyquist plot showed a low range sharp decrease of Z_im_ and then sharp increase of it till it finished for both before and after GCD. The point (Z_re_) at which Z_im_ value started to increase, was 7.63 Ω and 7.86 Ω, for before and after GCD, respectively. Before GCD this value is slightly higher than that of after GCD, explaining higher capacitance for fresh electrode than the electrode after GCD. These values are the lowest among the three modified electrodes, explaining the higher retention of GCD after 200 cycles compared to other two modified electrodes. Nyquist plot of Product-2@GCE (before GCD) showed a little bit different graph than that of Product-1@GCE. Here, the value of Z_im_ at high frequency region, decreased as like that of Product-1@GCE at Z_re_ = 8.52 Ω (Z1), then it traveled (very slight increase of the value of Z_im_, seemed almost like a tilted straight line) a path to Z_re_ = 11.48 Ω (Z2) and started to increase its Z_im_ value from middle to low frequency region. For after GCD, the value of Z_im_ decreased at Z_re_ = 18.37 Ω (Z3), then showed almost similar values till Z_re_ = 19.18 Ω (Z4) (which is lower than the value for before GCD), then started to increase the value of Z_im_. The separation between the Z1 and Z2 (2.96 Ω) is higher than the value between of Z3 and Z4 (0.81 Ω). This indicated slight detachment of electrode materials from the tip of GCE, which was higher at the beginning of GCD than that of after GCD. The value of Z4 is also higher than that of Z3, indicated lower value of retention after GCD. Product-3@GCE showed almost alike graph as Product-2@GCE. Here, the Nyquist plot started from Z_re_ = 5.39 Ω (Z5) (before GCD) and 9.72 Ω (Z7) (after GCD), then showed almost consistent values (very slight increase and/or decrease) till Z_re_ = 8.93 Ω (Z6) (before GCD) and 15.67 Ω (Z8) (after GCD), and then it started to increase to upward. The separation between Z5 and Z6 is 3.54 Ω, which is lower than the separation between Z7 and Z8 (5.95 Ω), explained the detachment of electrode material Product-3 started at the beginning of GCD and it continued after 200 cycles which could be happened due to the higher affinity of Product-3 for water than the inert Product-1 and 2. The remarkable higher value of Z_re_ after GCD indicated the lower retention of GCD for Product-3@GCE than other two electrodes [56]. The Bode plot of frequency dependent impedances (Fig 16(f)) showed the impedance values of Product-1@GCE for before (3.11) and after (3.10) GCD were almost similar and higher than other two electrodes, which were 2.01 (before), 1.99 (after) and 1.33 (before), 1.72 (after) for Product-2 and 3@GCE, respectively. All the electrodes showed low resistance. The highest phase angles obtained from the Bode plots of frequency dependent phase angle (Fig 16(g)) were 66.80 (before), 67.24 (after), 76.16 (before), 55.38 (after), 55.06 (before) and 37.53 (after) for Product-1, 2 and 3@GCE, respectively [56]. These values indicated the semiconductor property of Product-1, 2 and 3, which had been evaluated from GCD profile and also supported the Nyquist plot.

**Fig 17:**
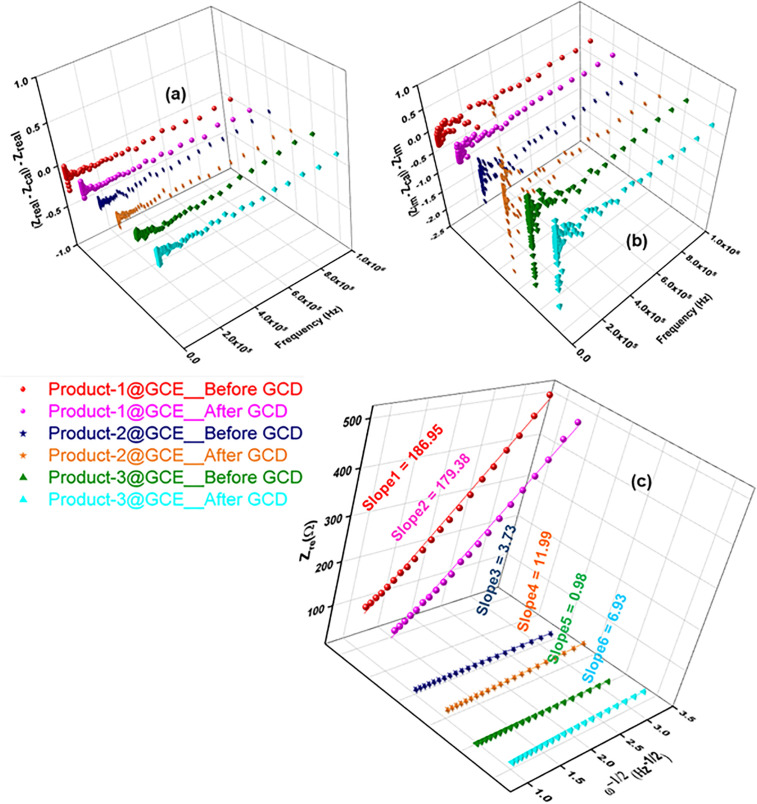
Error analysis of Nyquist plot; (a) Frequency vs. Z_real_ (b) Frequency vs. Z_im_ of Product-1, 2, and 3@GCE for before and after GCD, and (c) the relationship of corresponding Z_real_ and the reciprocal root square of angular frequency (ω^-^^1/2^).


The error analysis of Nyquist plot (Fig 17) stands as a proof for the validation of impedance fit parameters which obeys Kramers-Kronig relation [29]. The residual plots (Fig 17(a)) for Z_real_ over the frequency region completely satisfy the validation. The plot for -Z_im_ over frequency region (Fig 17(b)) completely satisfied at higher frequency but showed minor deviation for Product-2 and 3@GCE at lower frequency region. This relationship satisfied the linearity and stability criteria for impedance measurement. The relationship between the real part of impedance (Z_re_) and the reciprocal root square of angular frequencies (ω^-1/2^) (Fig 17(c)) can be expressed by following equation (7):

$$Z_{re}=R_{f}+R_{ct}+\sigma_{\omega }\omega^{-\frac{\text{1}}{\text{2}}}$$ (7)

Where, σ_ω_ is the Warburg impedance coefficient. ω^-1/2^ *vs.* Z_re_ graph (Fig 17(c)) of all electrodes for before and after GCD had been plotted, which depicts the relationship of resistance in lower frequency region of the cell according to the diffusion of electrons and H^+^ ions. The slopes gave the value of 186.95 and 179.38, 3.73 and 11.99, 0.98 and 6.93, for Product-1, 2 and 3@GCE at before and after GCD, respectively. The values of diffusion coefficient of H^+^ ion from electrolyte or electrons from the bulk electrode material could also be calculated according to following equation (8).


$$D=\text{0}.\text{5}{\left(\frac{RT}{AF\sigma_{\omega }C}\right)}^{\text{2}}$$ (8)

Where, D is the diffusion coefficient, R is gas constant, T is absolute temperature, F is Faraday’s constant, A is the area of electrode surface, and C is the molar concentration of H_2_SO_4_ solution (0.5 M in this case) [57,58]. The double layer capacitance, Q_dl_, could be calculated by following equations-


$$Z_{re}=R_{s}+R_{ct}+\text{2}\overset{\text{2}}{\underset{\omega }{\sigma }}Q_{dl}$$ (9)

$$\omega =\frac{\text{1}}{R_{ct}.Q_{dl}}$$ (10)

The calculated values of double layer capacitance (Q_dl_) is 1.473 × 10^-3^ and 1.654 × 10^-3^, 1.505 × 10^-2^ and 275.1, 21.15 × 10^-6^ and 13.33 × 10^-3^ F, for Product-1, 2 and 3 @GCE at before and after GCD, respectively. And those are for the charge transfer resistance (R_ct_) are 7.735 and 7.77, 7.76 and 24.16, 0.9749 and 10.82 Ω, respectively [59,60]. The exchange current density is given by the following equation-


$$i^{\text{0}}=\frac{RT}{nF}R_{ct}$$ (11)

Where, n is the no. of electron involved in the electrochemical reaction. The value of n is 3 in this case for all three electrode confirmed by the three set of anodic-cathodic peaks in CV data [61]. The calculated exchange current density of Product-1, 2 and 3@GCE at before and after GCD is 6.06 × 10^−2^ and 6.09 × 10^−2^, 6.08 × 10^−2^ and 1.89, 7.64 × 10^−3^, 8.4 A, respectively. All these values are very close to each other except the value for Product-3@GCE at after GCD. This is because of the affinity of Product-3 to water media. When the electrode was dipped in the 0.5 M H_2_SO_4_ aqueous media, it goes in the media creating the colorless solution to a blue colored one. It happened as soon as the electrode come in contact with the media which was very clear by bare eyes. Also the charging time of 1^st^ cycle of GCD of Product-3@GCE is much higher than the discharge time, which is the reason for thinning of electrode material at the beginning of GCD. After charging, lower amount of electrode material was there for discharging, promoting less active sites for reaction and huge number of free Product-3 in the water media for extensively high capacitance.

### Possible structures of prepared products

The experiments we have done was helpful to propose the possible structure of the compounds. For possible structure consideration, we analyzed the data sequentially -

1. From TGA (Fig 8), the products were considered as high temperature resistance compounds (Product-1 > Product-2 > Product-3). So, the products Might be or might not be polymers.
2. SEM (Fig 2) data showed, Product-1 and −2 having micro sized particle. Product-1 was seem a clear 3D crystals and very sharply distinguishable from Product-2 and −3. Unlike product-1, Product-2 seem like crystals having covered by some other materials, and Product-3 was confusing, it was look like Product-2 was covered by Product-3. This assumption was made because Product-2 was synthesized by heating Product-3 containing filtrate solution, while the color of the filtrate was similar as dry powder Product-3.
3. EDX (Fig 2) data showed all products were phosphomolybdates. The EDX spectra of Product-2, and −3 were alike, but different than Product-1.
4. FTIR (Fig 7) (Product −1) pattern was similar to kegging [10], and the other two were not like Product-1. Combining the previous data, Product-2 and −3 were other phosphomolybdates. The finger print region of all three products looked different.
5. Powder XRDs (Fig 3), in which prominent peaks are marked for the planes for already established unit clusters of Kegging [10] (for Product-1) and possibly hexamolybdate unit [62] cluster (for Product-2 and −3). The XRD pattern of Product-2, and −3 looked similar.
6. XPS data (Fig 4–6) indicated the oxidation states of Mn, elemental compositions and several bond natures. Combining the previous data, Product-1 was more logically considered as Kegging compound, and Product-2, and-3 were considered as polymer containing hexamolybdate unit cluster. The XPS spectra of Product-2, and −3 were alike and different than Product-1.
7. The CV (Fig 9–11, and 15) of product-1 is different but those for Product-2, and −3 were alike because their redox peaks were at the same position but current densities were different. For Product-1, high scan rates were applied and we got distinguishable clear peaks. Similar work was tried to apply for Product-2, and −3, but these two products showed broader peaks at high scan rates. As the scan rates were increased the peaks began to vanish. But they showed clear peaks at lower scan rates. From this experiment, it was noticed that the electrochemical behavior of Product-1 was different than other two, and Product-2, and −3 had similar CV profile.
8. For GCD profile (Fig 12–15), high current density was applied for Product-2, and −3. But low current density was applied for Product-1, because it showed lower capacitance as compared to other two. So, like CV, GCD also showed Product-2, and −3 had similar character but different than Product-1.
9. The Nyquist plot (Fig 16) of Product-1 also looked quite different than other two, while other two looked similar.

**Fig 18:**
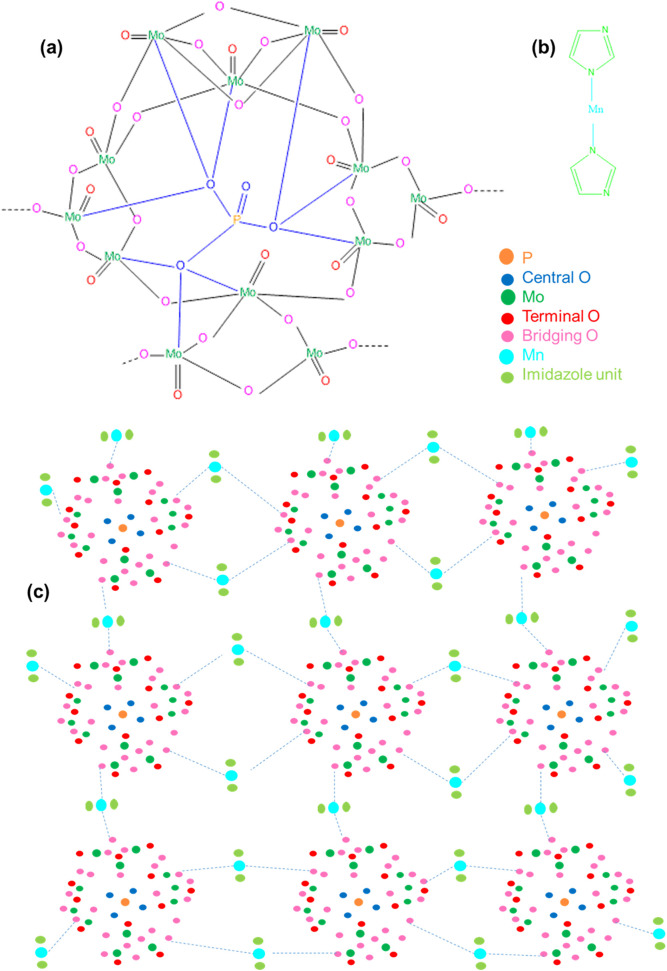
(a) Kegging cluster, (b) Bonding of Mn with imidazolium unit, and (c) Possible structure of Product-1.

**Fig 19:**
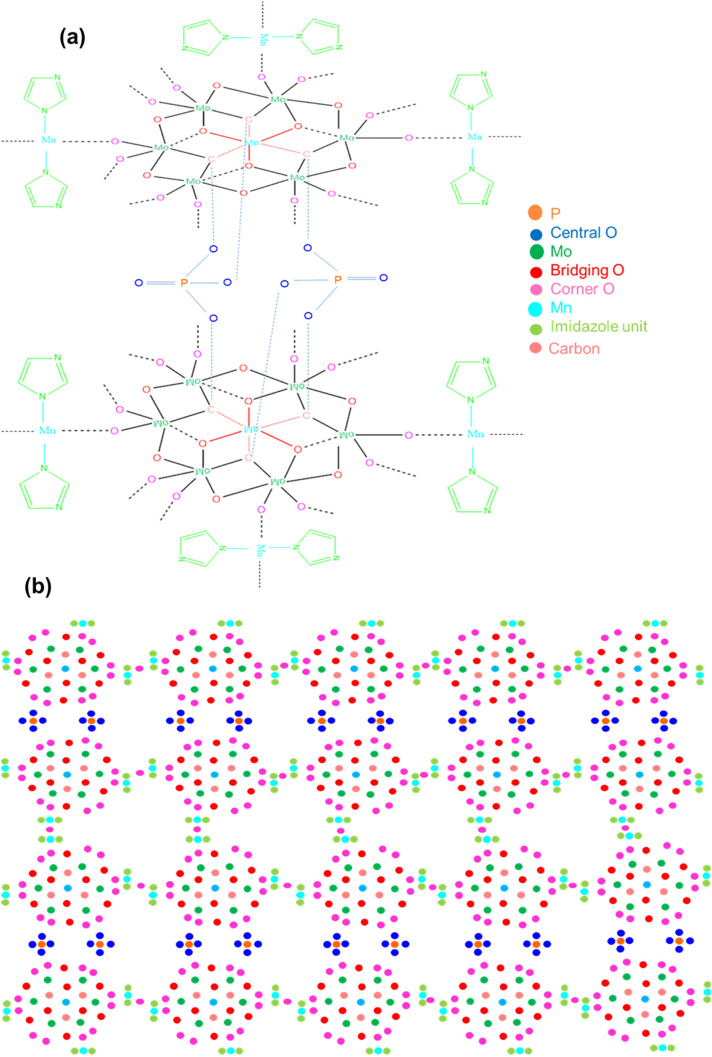
(a) Unit cell cluster, and (b) Possible structure of Product-2.

**Fig 20:**
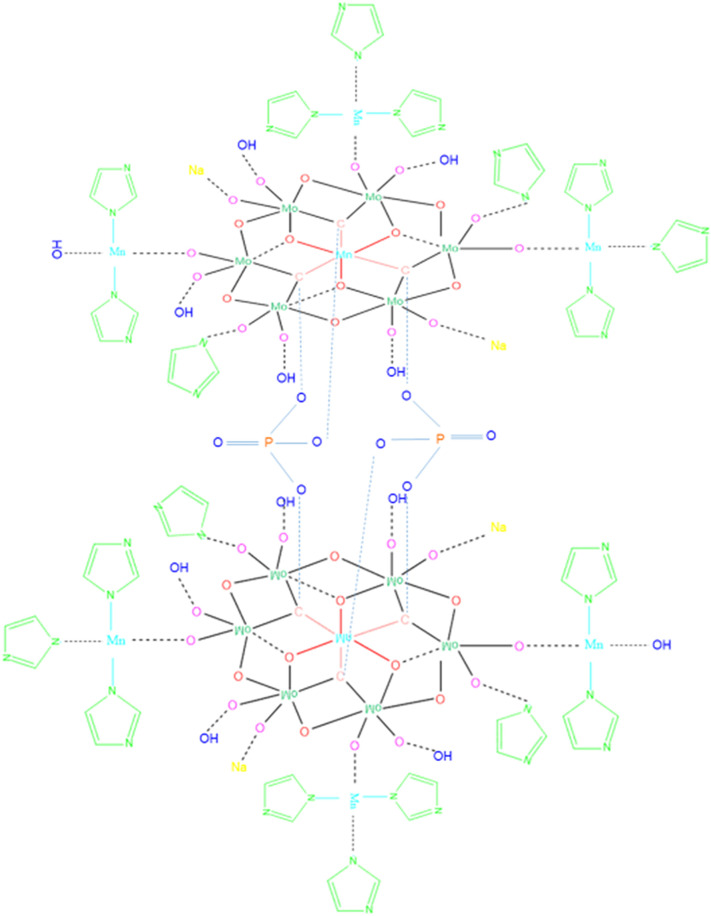
Possible structure for the major constituent of Product-3.

According to these characterizations and electrochemical evaluations, following possible structures were drawn for Product-1 (Fig 18) and Product-2 (Fig 19). Probable chemical formulas of Product-1, 2, and 3 were considered to be [Mn_x_(Imi)_y_{PMo_12_O_40_}], [Mn_x_(Imi)_y_{P_2_Mn_2_Mo_12_O_50_}] and [Mn_x_(Imi)_y_{P_2_Mn_2_Mo_12_O_50_(Imi)_4_Na_4_(OH)_8_}(OH)_z_], respectively. Because, Product-3 was not pure and was considered to be the combination of two or more by-products and/or electrolyte fragments produced by the main reaction, possible structure of the major constituent of it was drawn (Fig 20), which has a similarity with the unit cell cluster of Product-2, might have chemical formula [Mn_x_(Imi)_y_{P_2_Mn_2_Mo_12_O_50_(Imi)_4_Na_4_(OH)_8_}(OH)_z_], as commendable by the previous results and the literature [10,63]. These structures were not the exact structures but the probable structures of the products, based on stepwise evaluation and literature.

## Conclusion

A simple hydrothermal method was used to synthesize Kegging cluster consisted Mn-based phosphomolybdates, Product-1. The filtrate of this preparation was used to synthesize other two byproducts, happened to be Product-2 as main byproduct and Product-3 as remaining byproduct or a combination of remaining byproducts. All the products had distinct colors and were easily distinguishable by bare eyes. These products were characterize by SEM, EDS, XRD, XPS and TGA, and the result showed the existence of high temperature resistance Mn-based phosphomolybdates. Product-1 and 2 were water insoluble and Product-3 were water soluble. Product-2 was considered as the polymer or derivative of Product-3. Product-1, 2 and 3 were used to fabricate modified GCE electrodes to have Product-1, 2 and 3@GCE. All electrodes showed different reversible CV behavior with high capacitive contributions. Among these three electrodes, Product-3@GCE showed the highest current density as well as highest pseudocapacitive property. GCD showed at 2.0 Ag^-1^ current density, Product-1, 2 and 3@GCE showed specific discharge capacitance of 37.01, 380.45, and 635.37 Fg^-1^, respectively. GCD cycling of 200 cycles showed Product-1@GCE had comparatively higher retention as compared to Product-2 and 3@GCE. This work offered an environment friendly method for the synthesis of three phosphomolybdates by same reactants and single route having high pseudocapacitance.

## Supporting information

S1 Fig GCD cycling profile of Product-1@GCE for 1000 cycles at current density 0.4 Ag^-1^. (TIF)

S2 Fig GCD cycling profile of Product-2@GCE for 1000 cycles at current density 2.0 Ag^-1^. (TIF)

S3 Fig GCD cycling profile of Product-3@GCE for 1000 cycles at current density 2.0 Ag^-1^. (TIF)

S4 Fig GCD cycling profile of Carbon black@GCE at different current densities for 1000 cycling. (TIF)

S5 Fig Graphical abstract. (TIF)
