# Supplementary figures and images for: Mn-modified phosphomolybdates by hydrothermal route for pseudocapacitor application
Source: PLoS One. 2026 Apr 8;21(4):e0346559. doi: 10.1371/journal.pone.0346559 (PMC13061226; doi:10.1371/journal.pone.0346559)

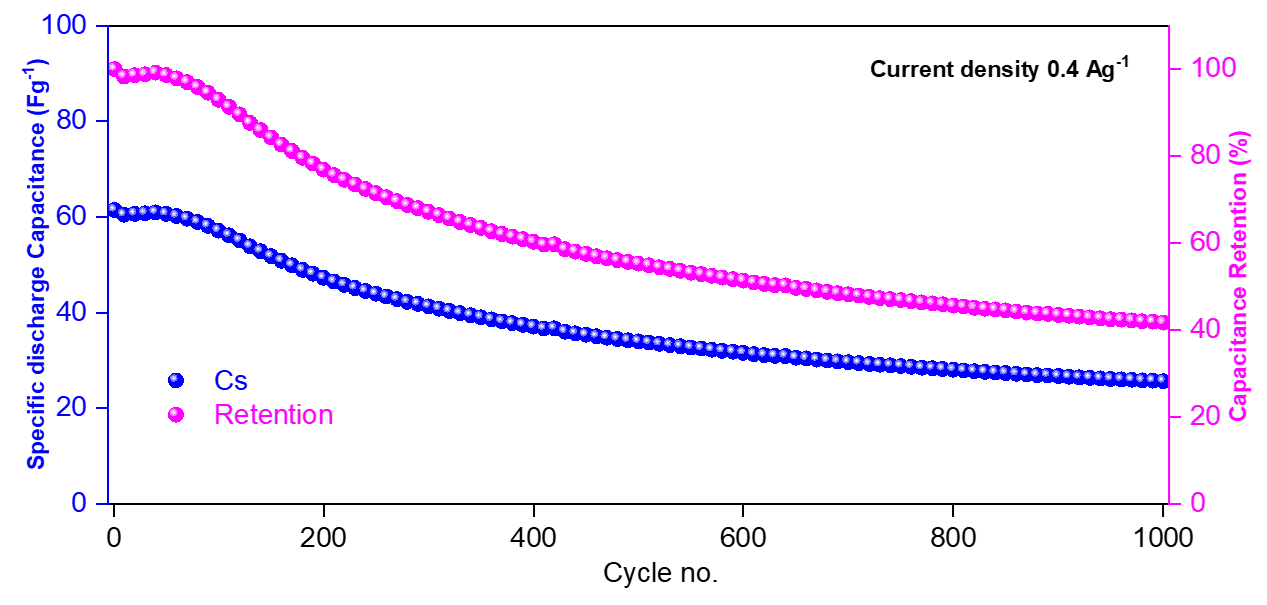

Supplement: S1 Fig — (TIF) [file pone.0346559.s001.tif]

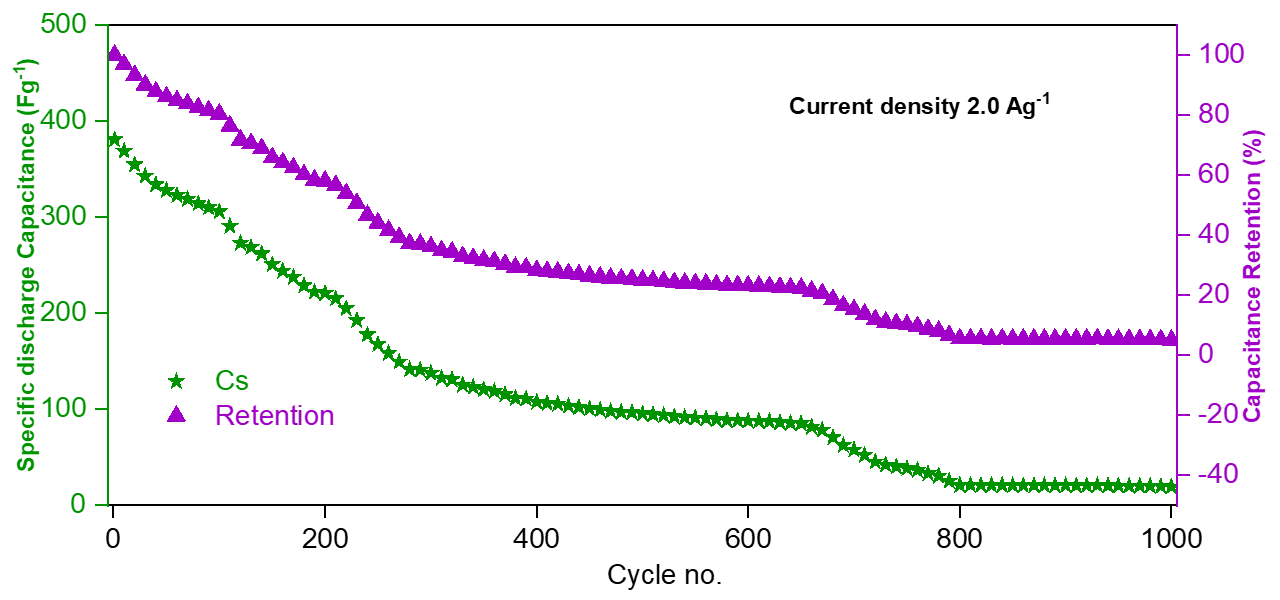

Supplement: S2 Fig — (TIF) [file pone.0346559.s002.tif]

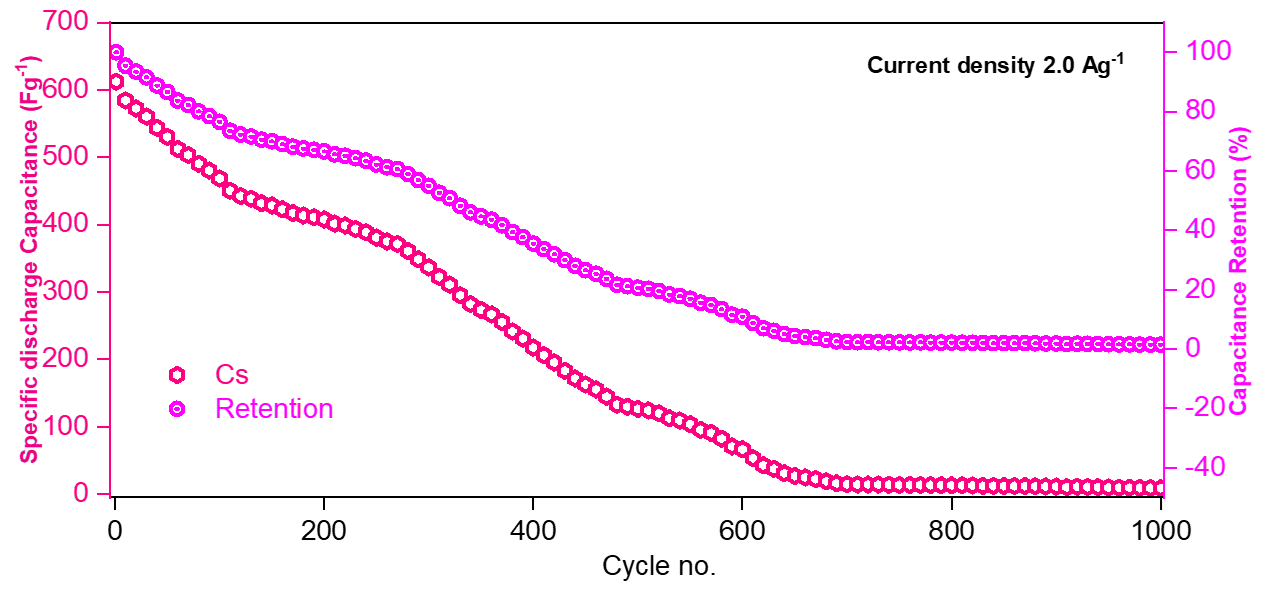

Supplement: S3 Fig — (TIF) [file pone.0346559.s003.tif]

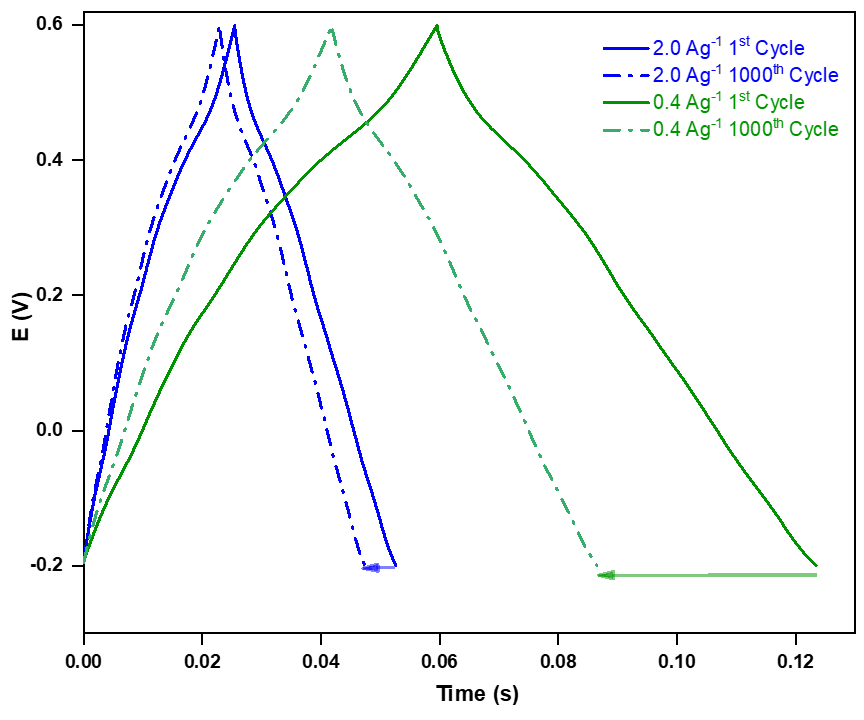

Supplement: S4 Fig — (TIF) [file pone.0346559.s004.tif]

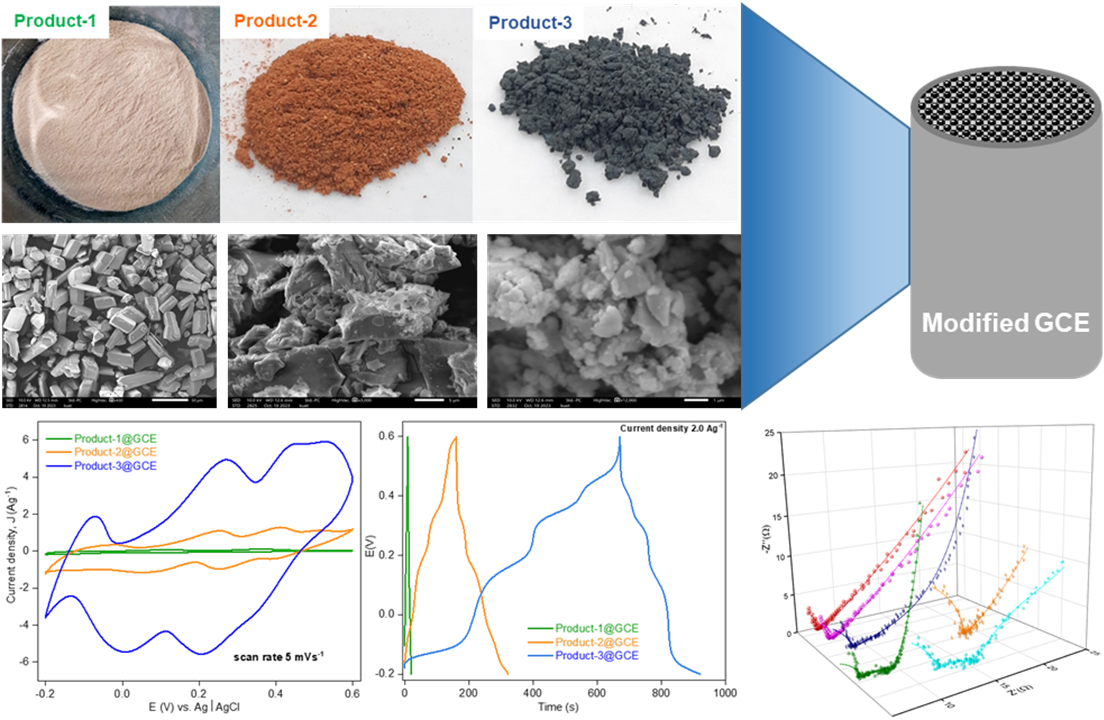

Supplement: S5 Fig — (TIF) [file pone.0346559.s005.tif]
